# Cadmium and High-Fat Diet Disrupt Renal, Cardiac and Hepatic Essential Metals

**DOI:** 10.1038/s41598-019-50771-3

**Published:** 2019-10-11

**Authors:** Jamie L. Young, Xiaofang Yan, Jianxiang Xu, Xinmin Yin, Xiang Zhang, Gavin E. Arteel, Gregory N. Barnes, J. Christopher States, Walter H. Watson, Maiying Kong, Lu Cai, Jonathan H. Freedman

**Affiliations:** 1 Department of Pharmacology and Toxicology, University of Louisville School of Medicine, Louisville, KY USA; 2 Department of Bioinformatics and Biostatistics, University of Louisville School of Public Health and Information Sciences, Louisville, KY USA; 3 Pediatric Research Institute, Department of Pediatrics, University of Louisville School of Medicine, Louisville, KY USA; 4 Department of Chemistry, University of Louisville, Louisville, KY USA; 5 Division of Gastroenterology, Hepatology and Nutrition, Department of Medicine, University of Pittsburgh, Pittsburgh, PA USA; 6 Division of Gastroenterology, Hepatology and Nutrition, Department of Medicine, University of Louisville School of Medicine, Louisville, KY USA

**Keywords:** Metals, Environmental impact, Outcomes research, Risk factors

## Abstract

Exposure to the environmental toxicant cadmium (Cd) contributes to the development of obesity-associated diseases. Obesity is a risk factor for a spectrum of unhealthy conditions including systemic metabolic dyshomeostasis. In the present study, the effects of whole-life exposure to environmentally-relevant concentrations of Cd on systemic essential metal distribution in adult mice fed a high-fat diet (HFD) were examined. For these studies, male and female mice were exposed to Cd-containing drinking water for >2 weeks before breeding. Pregnant mice and dams with offspring were exposed to Cd-containing drinking water. After weaning, offspring were continuously exposed to the same Cd concentration as their parents, and divided into HFD and normal (low) fat diet (LFD) groups. At 10 and 24 weeks, mice were sacrificed and blood, liver, kidney and heart harvested for metal analyses. There were significant concentration dependent increases in Cd levels in offspring with kidney > liver > heart. Sex significantly affected Cd levels in kidney and liver, with female animals accumulating more metal than males. Mice fed the HFD showed > 2-fold increase in Cd levels in the three organs compared to similarly treated LFD mice. Cadmium significantly affected essential metals levels in blood, kidney and liver. Additionally, HFD affected essential metal levels in these three organs. These findings suggest that Cd interacts with HFD to affect essential metal homeostasis, a phenomenon that may contribute to the underlying mechanism responsible for the development of obesity-associated pathologies.

## Introduction

Cadmium is a stable, persistent environmental toxicant used in the production of batteries, pigments, plastics and galvanized products. It is ranked number seven on the Agency for Toxic Substances and Disease Registry’s list of environmental chemical hazards and listed by the World Health Organization as “a major health concern”^1–3^. The average dietary intake of this metal ranges from 8–25 μg per day and it has a predicted half-life between 10–30 years^4,5^.The primary routes of exposure include ingestion of Cd containing foods or water and inhalation, specifically from cigarette smoke^5,6^.

Exposure to Cd is most commonly associated with nephrotoxicity, bone disease and cancer^4^. Urinary Cd levels are also positively correlated with increased hepatic necroinflammation, non-alcoholic fatty liver disease and non-alcoholic steatohepatitis in men, and necroinflammation in women^7^. Evidence suggests that Cd is also a risk factor for obesity-associated diseases because of its ability to alter systemic metabolism^8^. Links among Cd, obesity and disease in humans are controversial, as some epidemiological studies report no association^9–11^.

Mechanisms by which Cd contributes to a wide range of diseases have not been defined. Cadmium can mimic or replace essential metals in proteins^12^. For example, Cd displaces zinc (Zn) in many sulfur containing proteins leading to their dysfunction and subsequent disruption of numerous biological processes. Cadmium may also disrupt trace element homeostasis^13–15^. Disruption of metal homeostasis and pharmacokinetics is associated with metabolic abnormality such as obesity^12,16,17^. In fact, obesity is also associated with Zn deficiency, increased iron accumulation in the spleen and decreased iron level in the blood^14,18,19^. Compared to normal bodyweight controls, obese patients have increased plasma copper and manganese levels, which are associated with inflammation-induced disorders^19,20^. In addition, there are sex-specific differences in essential metal dyshomeostasis^15^.

Although epidemiological data suggest that Cd exposure is associated with a number of obesity-related diseases, there is a paucity of information regarding the interactive effects of Cd and HFD on the distribution of essential trace elements. To investigate this relationship, the effects of whole-life Cd exposure, at environmentally-relevant low-doses on trace element distribution in adolescent and adult mice were examined. Additionally, the role of obesity on trace element distribution was explored by feeding a HFD in combination with Cd. Additionally, sex differences in the distribution of these metals were examined.

## Results

### Effects of HFD and Cd exposure on body weight

**Figure 1:**
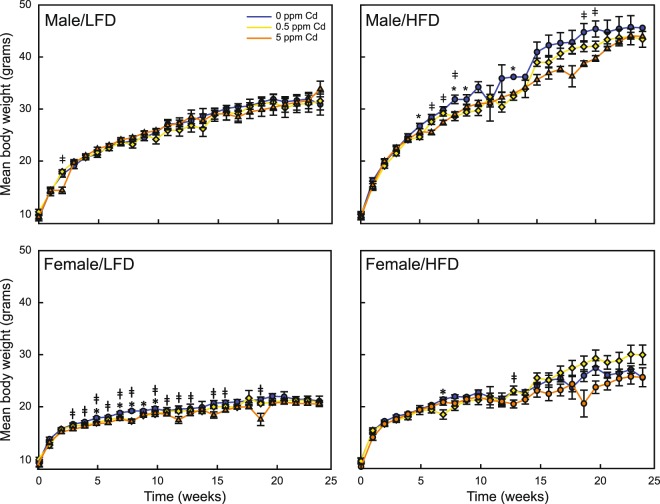
Effects of diet and toxicant exposure on body weight of F_1_ mice. F_1_ mice were treated as described in *Material and Methods* with body weight measured weekly. Body weight gain of male mice on a low fat (Male/LFD) or a high fat (Male/HFD) diet, and female mice on a low fat (Female/LFD) or a high fat (Female/HFD) exposed to drinking water containing 0 (blue line), 0.5 (yellow line) or 5.0 ppm (orange line) Cd is shown. Body weights are presented as means ± SEM for each group (n = 3–17). The asterisks indicate a significant difference (p < 0.05) between 0 and 0.5 ppm Cd. ^ǂ^ indicates a significant difference (p < 0.05) between 0 and 5 ppm Cd.

All animals gained weight as a function of time. There was no mortality or morbidity associated with any treatment group throughout the course of the study. Male mice fed HFD gained more weight compared to those fed LFD. Those exposed to 0 ppm Cd water and HFD gaining an average 0.42 grams more body weight per week compared to LFD fed male mice (Fig. 1). This trend was also seen in male mice exposed to 0.5 and 5 ppm Cd on HFD, with an average body weight gain of 0.44 and 0.45 grams per week, respectively, compared to LFD fed mice. Cadmium exposure did not alter body weight in male mice fed LFD. In contrast, male mice fed a HFD in combination with Cd gained less weight compared to 0 ppm Cd mice at multiple time points. HFD fed male mice exposed to 0.5 ppm Cd gained significantly less weight compared to 0 ppm Cd exposed mice after 5, 8, 9 and 13 weeks (p = 0.006, 0.007, 0.031 and 0.017, respectively). In addition, body weights of HFD-fed male mice at 6, 7, 8, 19, and 20 weeks of diet were significantly lower (p ≤ 0.001, 0.011, 0.007, 0.036 and 0.036, respectively).

Similar to the male cohort, female mice fed HFD gained more weight compared to those fed LFD but to a lesser degree (Fig. 1). Female mice exposed to 0 ppm Cd water on HFD gained an average of 0.13 grams more body weight per week compared to LFD female mice; ~70% less weight gain per week compared to males in the same Cd exposure group. Female mice exposed to 0.5 and 5 ppm Cd on HFD gained an average 0.38 and 0.27 grams more body weight compared to LFD fed mice, which is approximately 14 and 30 percent less weight gain, respectively per week compared to males in the same exposure group.

In contrast to males, Cd impacted bodyweight in females. Independent of diet, 0.5 ppm Cd exposure caused female mice to gain less weight during the first half of the study. After 5, 7, 8, 9 and 10 weeks of LFD, female mice exposed to 0.5 ppm Cd gained significantly less weight compared to 0 ppm Cd exposed animals (p = 0.020, 0.017, <0.001, 0.043 and 0.048, respectively). LFD fed female mice exposed to 5 ppm Cd also gained less weight compared to 0 ppm Cd animals. However, this lack of weight gain was observed throughout the study and not only in the first half as seen in the 0.5 ppm cohort. Cadmium exposure did not affect body weight gain in HFD fed female mice.

### Effects of HFD and Cd on water consumption

**Figure 2:**
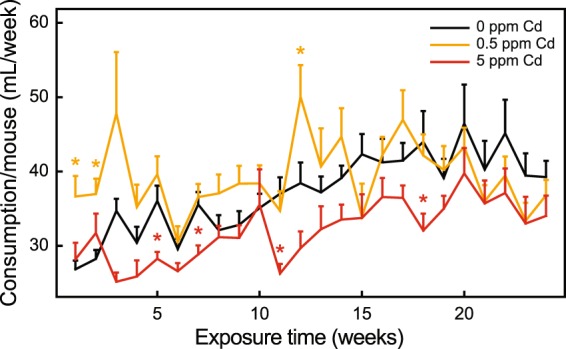
Effects of diet and toxicant exposure on water consumption in F_1_ mice. F_1_ mice were treated as described in *Material and Methods* with water consumption measured weekly. The cage mean per mouse and standard error of the mean (SEM) are presented for 0 (black), 0.5 (yellow) and 5.0 ppm Cd (red). The asterisks indicate that water consumption at the indicated time point was significantly different (p < 0.05) from 0 ppm Cd mice at the same time.

The initial analysis of water consumption considered time (weeks of exposure) and Cd concentration. At several time points, significant differences in water consumption between 0.5 or 5.0 ppm Cd and non-exposed mice were observed (Fig. 2). Additionally, water consumption increased until week 20 then declined in non-exposed mice. Water consumption by 0.5 ppm Cd exposure mice peaked at week 12 then declined. In general, consumption by 5.0 ppm Cd-exposed mice was the lowest, which peaked at week 20 (Fig. 2). Linear mixed effect models were applied to examine time and metal effects. Results showed that there were significant time and metal effect, and that their interaction also was significant (Table 1).

**Table 1:** Summary of ANOVA for water consumption.

Repeated two-way ANOVA	
Factor	p-value
Metal^a-0^	0.005
Time^b-0^	<0.001
Metal:Time^c-0^	0.001
**Repeated four-way ANOVA**	
Metal^a^	0.005
Time^b^	<0.001
Diet^c^	0.485
Sex^d^	0.06
Metal:Time*	0.004
Metal:Diet*	0.291
Time:Diet*	0.651
Metal:Sex*	0.768
Time:Sex*	0.225
Diet:Sex*	0.53
Metal:Time:Diet**	0.998
Metal:Time:Sex**	0.963
Metal:Diet:Sex**	0.165
Time:Diet:Sex**	0.786
Metal:Time:Diet:Sex***	0.911

^a-0^Indicates whether water consumption in F0 mice was significantly different among the three Cd exposure levels.

^b-0^Indicates whether water consumption in F0 mice was significantly different over time.

^c-0^Indicates whether the differences of water consumption between certain time points were significantly different among the three different Cd exposure levels.

^a^Indicates whether water consumption in F1 was significantly different among the three Cd exposure levels.

^b^Indicates whether water consumption in F1 was significantly different over time.

^c^Indicates whether water consumption in F1 was significantly different between HFDand LFD.

^d^Indicates whether water consumption in F1 was significantly different between female and male.

*Two-way interaction term indicates whether the difference of water consumption in F_1_ between two levels of one factor was significantly different among the different levels of the other factor; ** indicates three-way interactions and *** indicates four-way interactions.

Repeated four-way ANOVA (i.e., linear mixed effect models) were applied to determine time, metal, sex and diet effects (Table 1). Significant time, metal and their interactions were observed; however, sex and diet effects were not significant.

### Cadmium accumulation in blood and organs

Concentrations of Cd in blood, heart, kidney and liver were measured using ICP-MS. Cadmium concentrations were below the limits of detection (~0.15 ng/ml) in whole blood for all experimental conditions: sex, metal concentration, exposure time and/or diet (Suppl. File 1). In contrast, the metal was detected in the other tissues (Table 2, Suppl. File 1). Background levels of Cd were detected in the parental animals (F_0_), with the highest levels in the liver and kidney. Exposure to 0.5 and 5 ppm Cd resulted in a significant concentration-dependent increase in the metal (Tables 2 and 3). Metal accumulated to the greatest level in the kidney, with the smallest increase in heart. Additionally, there was a significant influence in Cd accumulation by sex; female mice had higher levels of metal in the three organs than males (Tables 2 and 3).

**Table 2:** Tissue Cd concentrations.

Treatment group					Concentration (n)^a^		
	Sex	Diet	Age (weeks)	Cadmium Concentration (ppm)	Heart	Kidney	Liver
F_0_	♂	NA	NA	0	1.2 ± 1.9(5)	12.2 ± 6.03(5)	24.1 ± 10.41(5)
		NA	NA	0.5	2.9 ± 1.92(5)	166.8 ± 39.03(5)	64.9 ± 3.25(5)
		NA	NA	5.0	48.9 ± 17.98(5)	3132.9 ± 698.07(5)	817.8 ± 237.1(5)
	♀	NA	NA	0	2.8 ± 0.88(4)	28.3 ± 15.76(5)	32.9 ± 5.68(5)
		NA	NA	0.5	20.7 ± 10.22(5)	1783.7 ± 390.54(5)	413.2 ± 159.87(5)
		NA	NA	5.0	244.3 ± 75.79(5)	14743.6 ± 4604.24(5)	7588.3 ± 2862.08(5)
F_1_	♂	LF	10	0	2.1 ± 2.6(8)	5 ± 6.99(8)	2.2 ± 3.16(8)
				0.5	4.1 ± 2.81(8)	217.5 ± 77.02(8)	78.8 ± 24.69(8)
				5.0	95.9 ± 44.13(7)	3624 ± 490.46(7)	1393.3 ± 226.02(7)
			24	0	0 ± 0(4)	7.2 ± 6.8(5)	3.2 ± 1.56(4)
				0.5	6.8 ± 3.15(4)	440.3 ± 111.31(4)	66.4 ± 29.49(4)
				5.0	98.6 ± 35.37(6)	6887.5 ± 1127.3(5)	953.1 ± 323.53(5)
		HF	10	0	2.2 ± 2.26(8)	5 ± 4.75(8)	4.1 ± 4.53(8)
				0.5	7.4 ± 3.14(7)	373.3 ± 110.76(7)	85.9 ± 40.87(7)
				5.0	152 ± 51.37(8)	5706.9 ± 1139.59(8)	2010 ± 856.03(8)
			24	0	3.4 ± 2.45(6)	7.1 ± 2.17(6)	4.3 ± 2.6(5)
				0.5	7.8 ± 3.36(6)	587.4 ± 198.99(6)	31.6 ± 11.6(6)
				5.0	147.3 ± 58.8(6)	11195.2 ± 1811.28(3)	737 ± 191.38(3)
	♀	LF	10	0	3.9 ± 2.08(6)	4.2 ± 6.63(6)	3 ± 3.8(6)
				0.5	7 ± 4.96(9)	309.8 ± 91.9(8)	118.2 ± 33.95(8)
				5.0	105.6 ± 15.18(10)	4909.1 ± 848.01(10)	2356.7 ± 446.26(10)
			24	0	3.2 ± 2.29(5)	2.6 ± 1.27(5)	2.5 ± 2.14(4)
				0.5	6.1 ± 5.01(5)	694.5 ± 98.56(5)	211.4 ± 15.37(5)
				5.0	111.5 ± 32.37(6)	10418.8 ± 1326.19(7)	2607.5 ± 516.08(7)
		HF	10	0	3.2 ± 5.82(7)	3.9 ± 2.74(7)	5.6 ± 7.19(7)
				0.5	8.5 ± 4.76(8)	506.6 ± 110.25(9)	129.6 ± 23.4(8)
				5.0	153.5 ± 56.55(10)	8117.5 ± 2624.51(10)	3006.4 ± 1384.27(10)
			24	0	4.5 ± 2.31(4)	11.8 ± 10(4)	3.2 ± 2.22(3)
				0.5	6.6 ± 3.1(5)	1661 ± 1119.82(5)	205.4 ± 147.81(5)
				5.0	256.6 ± 20.45(2)	18972.5 ± 4168.26(5)	4982.1 ± NA(1)

^a^Mean Cd concentration ± SD.

NA, not applicable; LF, low-fat diet; HF, high-fat diet.

**Table 3:** Summary of 2 and 4-way ANOVA for Cd.

Treatments	Heart	Kidney	Liver
	Pr(>F)		
	**F**_**0**_ **Generation**		
Cadmium concentration^a-0^	<0.001	<0.001	<0.001
Sex^b-0^	<0.001	<0.001	<0.001
Cadmium concentration: Sex^c-0^	<0.001	<0.001	<0.001
**Residuals**	—	—	—
	**F**_**1**_ **Generation**		
Cadmium concentration^a^	<0.001	<0.001	<0.001
Sex^b^	0.303	<0.001	<0.001
Diet^c^	<0.001	<0.001	0.018
Exposure time^d^	0.592	<0.001	0.286
Cadmium concentration: Sex*	0.495	<0.001	<0.001
Cadmium concentration: Diet*	<0.001	<0.001	0.002
Sex: Diet*	0.685	0.004	0.535
Cadmium concentration: Exposure time*	0.284	<0.001	0.724
Sex: Exposure time*	0.217	<0.001	0.006
Diet: Exposure time*	0.282	<0.001	0.769
Cadmium concentration: Sex: Diet**	0.602	0.061	0.557
Cadmium concentration: Sex: Exposure time**	0.109	<0.001	0.001
Cadmium concentration: Diet: Exposure time**	0.187	<0.001	0.932
Sex: Diet: Exposure time**	0.067	0.094	0.057
Cadmium concentration: Sex: Diet: Exposure time***	0.029	0.238	0.006
Residuals	—	—	—

^a-0^Indicates whether Cd concentration in F0 was significantly different among the three Cd exposure levels.

^b-0^Indicates whether Cd concentration in F0 was significantly different between male and female.

^c-0^Indicates whether the difference of Cd concentration between male and female was significantly different among the three different Cd exposure levels.

^a^Indicates whether Cd concentration in F1 was significantly different among the three Cd exposure levels.

^b^Indicates whether Cd concentration in F1 was significantly different between male and female.

^c^Indicates whether Cd concentration in F1 was significantly different between HFD and LFD.

^&^Indicates whether Cd concentration in F1 was significantly different between 10 and 24 weeks exposure.

^d^Two-way interaction term indicates whether the difference of Cd concentration in F1 between two levels of one factor was significantly different among the different levels of the other factor; ** indicates three-way interactions and *** indicates four-way interactions.

Similar to F_0_ animals, there were significant concentration-dependent increases in Cd levels in treated offspring (F_1_). The level of accumulation in kidney was triple the concentration in liver and almost ten-fold greater than the heart (Tables 2 and 3). Sex significantly affected Cd levels in kidney and liver, with female animals accumulating more metal than males. There was not a significant sex difference in the heart. Kidney showed significant accumulation of metal as a function of exposure time (10 vs 24 week) (Tables 2 and 3). These results suggest that the metal storage capacity of the heart and liver may become saturated.

High fat diet significantly affected Cd accumulation in the three organs. Mice fed the HFD showed >2-fold increase in Cd levels compared to similarly treated LFD mice (Tables 2 and 3). Two-way ANOVA for concentration and diet indicated significant interactions for the three organs (Table 3).

Two- and four-way ANOVA of Cd in heart, liver and kidney identified multiple significant interactions among the four experimental conditions (Table 3). ANOVA indicated that the level of Cd in the kidney was most affected by combinations of these conditions. These observations support a model where obesity or associated metabolic disorders may affect Cd toxicity by influencing kidney metabolism.

### Effect of Cd exposure on essential metal levels in blood and organs

ICP-MS measures concentration of metals with Z numbers between 9 (Be) and 208 (Pb) (Suppl. File 1). Additional analyses focused on the effects of Cd (concentration and exposure time), diet and sex on the following essential metals: ^23^Na, ^24^Mg, ^39^K, ^44^Ca, ^55^Mn, ^57^Fe, ^59^Co, ^65^Cu, ^66^Zn, ^82^Se, and ^95^Mo. (Summaries of metal concentrations in blood, heart, liver and kidney and associated ANOVAs for F_0_ and F_1_ mice can be found in Suppl. Tables 1–11).

Although levels of Cd significantly increased in the heart, neither metal exposure nor diet significantly affected levels of the essential metals in this organ (Table 4). Significant metal-independent sex differences in cardiac levels of Na, Co and Mo were observed.

**Table 4:** Essential elements affected by Cd exposure and/or HFD*.

	Cadmium	Diet	Cadmium:Diet
Blood	Mg, K, Fe, Cu, Zn	Cu	—
Liver	Mg, K, Mn, Zn, Mo	Mg, K, Mn, Fe, Co, Cu	Fe, Co
Kidney	Na, Mg, K, Ca, Mn, Fe, Cu, Zn, Se, Mo	Mn, Fe, Co, Mo	—
Heart	—	—	—

*Significant effect (p < 0.05) observed based on ANOVA.

Cadmium significantly affected the levels of Mg, K, Fe, Co, Cu and Zn in blood, either as a function of metal concentration and/or exposure time (Table 4). Magnesium showed a concentration dependent decrease. Additionally, K, Fe, Cu, and Zn levels decreased following exposure to 0.5 ppm Cd, compared to 0 ppm Cd, and then remained constant or increased with 5 ppm exposures, compared to 0.5 ppm. There were also metal-independent, sex differences in Na, Mg, Fe, Cu, Zn and Mo in F_0_ and/or F_1_ mice. HFD did not significantly affect the levels of the essential metals, with the exception of Cu.

In kidneys of F_1_ mice, which accumulated the highest levels of Cd, metal treatment significantly affected concentrations of all of the measured essential metals as functions of metal concentration and/or exposure time (Table 4). In F_0_ mice, 5.0 ppm Cd treatment resulted in a significant increase in Zn. Treatment with 0.5 ppm Cd caused significant decreases in Na, Mg, K, Ca, Fe, and Co in F_1_ mice. Levels of Mg, K, Mn, Zn and Mo were usually higher at 0.5 ppm compared to 0 ppm Cd mice, or those exposed to 5 ppm Cd. There were metal-independent sex differences in the levels of Na, Ca, Mn, Fe, Cu, and Mo in F_0_ and/or F_1_ mice. Additionally, in the absence of Cd, HFD significantly affected the levels of Mn, Fe, Co and Mo. Both Fe and Co were significantly lower in HFD mice compared to LFD animals. At 5.0 ppm Cd, Zn and Cu levels were the highest in the HFD group. Additionally, Se and Mo had the highest values at 5.0 ppm Cd in the 24 week HFD group.

The levels of Fe and Zn significantly changed with Cd exposure in the livers of F_0_ mice. Additionally, there were significant differences by sex for Na, Mg, Cu and Mo (Suppl. Tables 1, 2, 9 and 11).

The concentrations of Na, Mg, K, Mn, Zn and Mo in the liver of F_1_ animals were dependent on the Cd exposure level and time; where their levels were generally higher at 0.5 ppm compared to the 0 and 5 ppm exposure groups. Magnesium, K, Mn, Fe, Co and Cu levels were affected by diet, independent of metal exposure. Specifically, (a) the levels of Mg and K in 24 week LFD fed mice were significantly higher than HFD fed mice; (b) the level of Mn in LFD female mice was significantly lower than HFD female mice; and (c) the levels of Fe and Co were significantly higher in LFD mice compared to those fed HFD. Significant metal-independent sex differences were also observed in Na, K, Mn, Fe, Co, Cu and Zn (Suppl. Tables 1, 3, 5–9).

## Discussion

To investigate the relationship among Cd exposure, obesity and metal homeostasis, mice were exposed to low concentrations of Cd. Many of the previous studies used environmentally irrelevant, toxic doses of Cd^21–25^. The lack of overt toxicity associated with 5 and 0.5 ppm Cd exposures was confirmed by histopathological analyses of heart, liver and kidney (Data not shown).

Ingestion is the primary route of Cd exposure in the general, non-smoking population. Therefore, mice were given *ad libitum* access to Cd containing drinking water. There was a general trend of reduced water consumption as a function of time for 5 ppm Cd exposure group, compared to 0 ppm Cd exposed mice (Fig. 2). This observation is similar to that previously reported^26,27^. Although daily Cd consumption decreased during the course of the study, Cd concentrations in the heart, liver and kidney increased in both male and female F_1_ mice (Table 2).

Cadmium, a cumulative toxicant with a biological half-life of >20 years, is primarily stored in the liver and kidney^28^. Non-exposed F_0_ and F_1_ mice had detectable levels of Cd in the heart, liver and kidney. The levels of Cd in the liver and kidney of F_0_ mice were 2-10-times greater than non-exposed F_1_ animals. This may be a consequence of the F_0_ animals being fed standard laboratory chow prior to arriving in the laboratory^29^. F_1_ mice were only fed a metal-reduced, purified diet.

Levels of Cd in liver and kidney of F_1_ mice significantly increased as functions of time and concentration. Similar to previous reports, the greatest accumulation of Cd was found in the kidney^27,30,31^. Approximately three times more Cd accumulated in the kidney compared to the liver, a phenomenon previously observed in rodent models^31,32^. Independent of exposure concentration, Cd accumulation in the liver decreased or plateaued between 10 and 24 weeks in F_1_ mice (Table 2). Similar responses were observed in mice exposed to 100 ppm CdCl_2_ (~61 ppm Cd) in drinking water for 1, 4, 8, 16 and 23 weeks. In this study, hepatic Cd increased until 16 weeks and then began to decrease. These observations support a model of Cd homeostasis where hepatic Cd is released into the blood where it is taken up and terminally stored in the kidney^33^. Alternatively, the metal may be excreted into the bile and reabsorbed into the circulation via the gut^34^.

Cadmium accumulated in the heart in a concentration dependent manner and plateaued between 10 and 24 weeks of exposure. A similar mechanism of Cd homeostasis between the heart and kidney may also be responsible for this phenomenon. Cadmium exposure is positively correlated with increased incidences of cardiovascular diseases^35–37^. These correlations are based on blood and/or urine Cd levels^38^. There is a paucity of data implicating cardiac Cd levels with negative cardiac outcomes. Future studies will specifically address this gap in our understanding the relationship between cardiac Cd and cardiovascular disease.

It has been reported that Cd accumulation in blood occurs in a time and concentration dependent manner, but tends to reach a plateau or decrease between 3–5 months of exposure^26,39,40^. In contrast, we found that Cd concentrations in the blood were below the limits of detection, which may be a consequence of differences in experimental design. In the present study, measurements were made after 10 and 24 weeks of exposure, at which point the half-life of Cd in blood may decrease due to more efficient uptake and storage in other organs. Additionally, low concentrations of Cd were used, while in other studies higher doses were used and measurements made at more acute time points^26,32^.

Human and animal studies report that females have a greater Cd body burden^41^. This sexual dimorphism in Cd accumulation was also observed in F_0_ and F_1_ animals. F_0_ dams had significantly higher levels of Cd in all three organs compared to their male counterparts. F_1_ female mice accumulated more metal in liver and kidney, but not in the heart, than males (Tables 2 and 3). Similarly, Dip (2017) found no statistical difference in Cd accumulation in the hearts of males and females^42^. The sexual dimorphism in Cd accumulation may be related to the expression of DMT1, the non-specific divalent metal transporter, which mediates the absorption of Cd in the small intestine^43,44^. The density of DMT1 transporters increases in females under conditions of Fe depletion (see below) and pregnancy^45–47^.

The absorption of Cd through the gastrointestinal tract is influenced by nutritional status^28^. F_1_ mice fed HFD accumulated significantly higher levels of metal in the three organs (Tables 2 and 3). This HFD response may be a consequence of a general dysregulation of metal homeostasis. Obesity is associated with dysregulation of Mg, Fe, Zn, Cu and Ca homeostasis^48,49^. Similarly, F_1_ mice fed HFD for 24 weeks showed altered levels of these metals in blood, liver and kidney; as well as K, Co, Na, Mn and Mo. Additionally, independent of diet, Cd exposure significantly affected essential metal homeostasis (Table 4, Suppl. Tables 1–11).

Obesity and Cd exposure are associated with anemia and Fe deficiency^50–53^. Iron was one of the essential metals affected by both Cd and HFD in F_1_ mice. Iron levels decreased in blood and kidney, with a significant metal:diet interaction in liver (Table 4). Additionally, HFD alone significantly reduced Fe levels in the liver and kidney of both male and female mice. HFD-induced Fe deficiency may be due to low intake, reduced absorption and/or chronic inflammation^54,55^. Fe deficiency may also be the result of Cd competition with the DMT1 transporter. Interestingly, a Cd-induced Fe deficiency would increase the expression of DMT1 that would lead to further increases Cd absorption^4,56^. A sexual dimorphism was observed in the current study where females exposed to Cd had less Fe in their blood and more Cd in kidney and liver. This is similar to that previously reported^6,43^.

**Figure 3:**
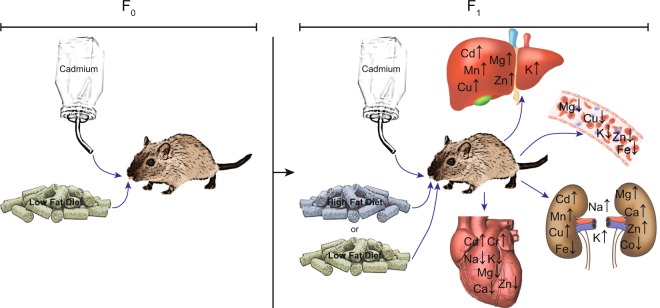
Summary results on the effects of Cd and diet on essential metal homeostasis. Mice (parents, F_0_ and offspring, F_1_) were exposed to Cd and/or HFD as diagrammed in Fig. 4. After 10 and 24 weeks liver, blood, kidney and heart were collected and Cd and essential metals measured by ICP-MS. Metals whose levels significantly changed under any condition are presented.

In summary, whole life exposure to low doses of Cd in combination with HFD significantly affected essential metal homeostasis (Fig. 3). In addition, sex differences in Cd and essential metal distribution were observed. The disruption of essential metal homeostasis may contribute to processes underlining differences in the development of obesity-associated diseases between males and females.

The mechanism by which Cd and/or obesity affect essential metal homeostasis is currently unknown. The exposure scheme used in this study, pre-conception (F_0_) to sacrifice (F_1_), supports a model where Cd could directly affect offspring or promote an epigenetic response. Exposure to Cd induced changes in DNA methylation, histone modification and microRNAs^57^. Treatment of rats and mice with low dose Cd causes hypermethylation of hepatic genes^58^. Additionally, maternal Cd exposure is associated with epigenetic changes in offspring^59,60^. Interestingly these changes show sex specificity^61^. High-fat diet-induced obesity could also induce epigenetic changes in the offspring. Literature surveys report that the expression of dozens of genes is modified by obesity^62,63^.

In summary, the results from this study will serve as the basis for future studies focused on the pathophysiological consequences of Cd exposure and obesity on diabetes, and hepatic, renal and cardiac function.

## Methods

### Animals and exposures

Six week old male and female C57BL/6J mice were purchased from Jackson Laboratory (Bar Harbor). Mice were maintained on a 12 h light/dark cycle at 25 °C in a pathogen-free AAALAC-accredited facility. All procedures were approved by the University of Louisville’s Institutional Animal Care and Use Committee and experiments were performed in accordance with relevant guidelines and regulations. One week after the animals arrived, diets were changed from standard laboratory chow to AIN-76A purified diet (Envigo TD 160377) to limit the confounding effects of metal contamination found in standard chow^29^. Food and deionized water were provided ad libitum. Water consumption was recorded weekly.

**Figure 4:**
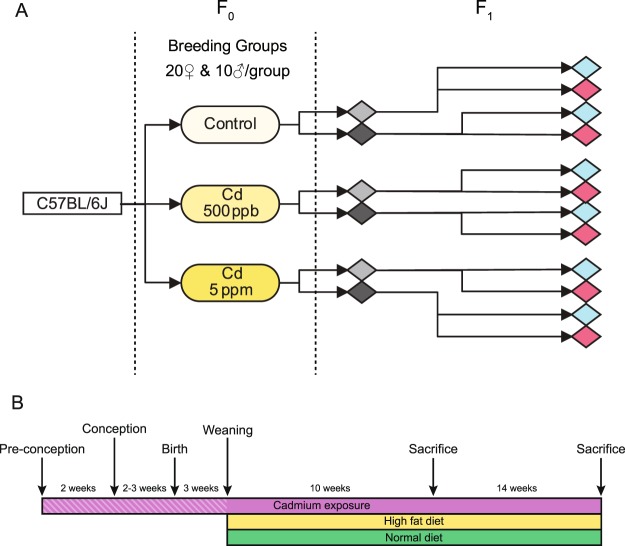
Multigenerational exposure to Cd in conjuncture with diet. Adult male and female C57BL/6J mice on defined, low-fat diets were exposed to drinking water containing 0, 0.5 or 5 ppm Cd for >2 weeks before being established into eight breeding triplets (F_0_). F_0_ mice (n = 8 ♂, n = 16♀ for each concentration) were continuously exposed to Cd during pregnancy. The offspring (F_1_), four male and four female mice from each triplet were exposed to the same toxicants as their parents after weaning. At weaning, male (*blue*) and female (*pink*) offspring were fed either a low- (n = 8; *light gray diamonds*) or high-fat diet (n = 8; *dark gray diamonds*), respectively for 10 or 24 weeks. *Hatched lines*; F_0_ mice.

Cadmium exposure for parental mice (F_0_) began at 10 weeks of age. Cadmium containing drinking water (0, 0.5 or 5 ppm (final concentration)) was prepared from stock solutions of CdCl_2_ • 5H_2_O (Alfa Aesar), in deionized water and stored at −80 °C. Based on a survey of the literature, 5 ppm Cd is one of the lower concentrations of Cd tested with results supportive of metabolic syndrome phenotypes. Therefore 5 ppm Cd was used as a positive control, as well as a ten times lower concentration of 0.5 ppm, which are approximately 1% and 0.1% of the Cd LD_50_^3^. Additionally, the lowest concentration of Cd tested is within the range found in polluted waters^28,64,65^. At 12 weeks of age mice were placed into breeding triplets (1 male to 2 females) within each Cd exposure group (Fig. 4). After weaning, offspring (F_1_) were continuously exposed to the same concentration of Cd as their parents until sacrifice. Additionally, offspring were fed either a low-fat (Envigo TD 160377 - 13% fat, Madison, WI) or high-fat (Envigo TD 09682 – 42% fat, Madison, MI) diet (See Suppl. File 2 for diet composition). During metal and diet exposure, weights of individual animals were measure weekly (Fig. 1). Mean weight change per week was calculated from the average differences within an exposure group in weight between 0 and 24 weeks of individual mice divided by 24.

Offspring were sacrificed 10 or 24 weeks after weaning. Mice were anesthetized with ketamine/xylazine and blood was collected from the vena cava prior to sacrifice via exsanguination. Blood samples were centrifuged and citrated plasma stored at −80 °C until analyzed. Heart, liver and kidney were harvested from each mouse. Portions of each organ were snap-frozen in liquid nitrogen for metals analyses.

### Metal analysis

Whole blood, liver, kidney and heart samples were digested following incubation in 70% nitric acid at 85 °C for 4 h. Samples were then cooled to room temperature, centrifuged to remove undigested debris and then diluted to 2% nitric acid (final concentration) with Milli-Q deionized water. Element quantification was performed using an X Series II quadrupole inductively coupled plasma mass spectrometry (ICP-MS) (Thermo Fisher Scientific) equipped with an ESI SC-2 autosampler (Elemental Scientific, Inc.) for sample injection. During sample injection, internal standards including Bi, In, ^6^Li_,_ Sc, Tb and Y (Inorganic Ventures) were mixed with each sample for instrument calibration. Each sample was analyzed three times. The ICP-MS was operated in CCT-KED mode with operation parameters as follows: forward power 1,404 W; sampling depth 150 mm; nebulizer gas flow 0.84 min^−1^; auxiliary gas flow 0.80 min^−1^; coolant gas flow 13.0 min^−1^; sweeps 130; acquisition duration 30 s; dwell time 5 ms; analogue detector voltage 1,850 V; PC detector voltage 2,900 V.

### Statistical analyses

Descriptive statistics for parental mice (F_0_) are summarized as mean, standard deviation (SD), and sample size (i.e., mean ± SD (n)), stratified by sex and Cd concentration. Descriptive statistics for offspring mice (F_1_) are summarized as mean ± SD (n) stratified by sex, diet (LF vs. HF), exposure time (10 vs 24 weeks) and Cd concentration. Two-way ANOVA with interaction was applied to examine whether Cd concentration and sex are significant for each metal concentration in parental mice. Four-way ANOVA with interactions was applied to examine whether sex, diet, exposure time and Cd exposure are significant for each metal in offspring mice. The p-values resulting from the ANOVAs are reported.

The main effect or interaction is significant if the corresponding p-value was less than 0.05. For example, the small p-values in Table 3 for F_0_ generation indicate that (a) the Cd concentrations in heart, kidney or liver were significantly different among the three Cd exposure levels; (b) the concentrations were significantly different between male and female animals and (c) the difference in metal concentration between F_0_ male and female mice was significantly different among the three Cd doses.

In F_1_ animals a significant Cd exposure effect indicates that the metal was significantly different among the three Cd exposure levels; significant sex effect indicates that metal concentration was significantly different between male and female mice; significant diet effect indicates that metal concentration was significantly different between HFD and LFD fed mice and a significant exposure time effect indicates that metal concentration was significantly different between 10 and 24 weeks exposure groups. Two-way interaction term indicates whether the difference of metal concentration in F_1_ between two levels of one factor was significantly different among the different levels of the other factor. Three-way interactions and four-way interactions were further interpreted due to their complexity.

Water consumption is summarized by mean ± SD. Repeated ANOVA is applied to determine if water consumption changes over time, and if the effects of sex, diet, and Cd concentrations are significant. Body weight is also summarized as means ± standard error mean (SEM; n = 3–10). Four-way ANOVA was used for the determination of statistical significance of sex, diet, Cd concentration, and exposure time.

## Supplementary information

Supplementary Information

Supplementary Dataset 1
