# Publisher Correction: Cadmium and High-Fat Diet Disrupt Renal, Cardiac and Hepatic Essential Metals

**DOI:** 10.1038/s41598-020-58517-2

**Published:** 2020-02-10

**Authors:** Jamie L. Young, Xiaofang Yan, Jianxiang Xu, Xinmin Yin, Xiang Zhang, Gavin E. Arteel, Gregory N. Barnes, J. Christopher States, Walter H. Watson, Maiying Kong, Lu Cai, Jonathan H. Freedman

**Affiliations:** 1 Department of Pharmacology and Toxicology, University of Louisville School of Medicine, Louisville, KY USA; 2 Department of Bioinformatics and Biostatistics, University of Louisville School of Public Health and Information Sciences, Louisville, KY USA; 3 Pediatric Research Institute, Department of Pediatrics, University of Louisville School of Medicine, Louisville, KY USA; 4 Department of Chemistry, University of Louisville, Louisville, KY USA; 5 Division of Gastroenterology, Hepatology and Nutrition, Department of Medicine, University of Pittsburgh, Pittsburgh, PA USA; 6 Division of Gastroenterology, Hepatology and Nutrition, Department of Medicine, University of Louisville School of Medicine, Louisville, KY USA

Correction to: *Scientific Reports* 10.1038/s41598-019-50771-3, published online 11 October 2019

The original PDF version of this Article contained missing images in Figure 3. This has now been corrected in the PDF; the HTML version of the paper was correct from the time of publication.

