# Supplementary material for: Cadmium and High-Fat Diet Disrupt Renal, Cardiac and Hepatic Essential Metals
Source: Sci Rep. 2019 Oct 11;9:14675. doi: 10.1038/s41598-019-50771-3 (PMC6789035; doi:10.1038/s41598-019-50771-3)
Supplement: Supplementary file 1 — Supplementary Information [file 41598_2019_50771_MOESM1_ESM.pdf]

CADMIUM AND HIGH-FAT DIET DISRUPT RENAL, CARDIAC AND HEPATIC ESSENTIAL METALS

Jamie L. Young [1], Xiaofang Yan [2], Jianxiang Xu [3], Xinmin Yin [4], Xiang Zhang [1,4], Gavin E. Arteel [1, 5], Gregory N. Barnes [3], J. Christopher States [1], Walter H. Watson [1], Maiying Kong [2], Lu Cai [1,3] * and Jonathan H. Freedman [1] *

From the [1] Department of Pharmacology and Toxicology, University of Louisville School of Medicine; [2] Department of Bioinformatics and Biostatistics, University of Louisville School of Public Health and Information Sciences; [3] Pediatric Research Institute, Department of Pediatrics, University of Louisville School of Medicine; [4]Department of Chemistry, University of Louisville and [5] Division of Gastroenterology, Hepatology and Nutrition, Department of Medicine, University of Pittsburgh.

***Corresponding authors**:

Dr. Jonathan Freedman, Department of Pharmacology and Toxicology, University of Louisville, 500 S. Preston Street, HSC-A, Louisville, KY 40202, USA. Email: jonathan.freedman@louisville.edu;

Dr. Lu Cai, Pediatric Research Institute, Department of Pediatrics, University of Louisville School of Medicine, 570 S. Preston St., Louisville, KY 40202, USA. Email: lu.cai1@louisville.edu

Low Fat Control Diet (Green)

	g/Kg
Casein	195.0
DL-Methionine	3.0
Sucrose	120.0
Corn Starch	432.9
Maltodextrin	100.0
Anhydrous Milkfat	37.2
Soybean Oil	12.8
Cellulose	50.0
Mineral Mix, AIN-76 (170915)	35.0
Calcium Carbonate	4.0
Vitamin Mix, Teklad (40060)	10.0
Ethoxyquin, antioxidant	0.01
Green Food Color	0.1

Selected Nutrient Information

Item	*% by Weight*	*% kcal from*
Protein	17.3	19.1
Carbohydrate	61.3	67.9
Fat	5.2	13

Kcal/g = 3.6

42% Fat Calories Diet (Western, Blue)

	g/Kg
Casein	195.0
DL-Methionine	3.0
Sucrose	341.5
Corn Starch	150.0
Maltodextrin	100.0
Anhydrous Milkfat	210.0
Cholesterol	1.5
Cellulose	49.9
Mineral Mix, AIN-76 (170915)	35.0
Calcium Carbonate	4.0
Vitamin Mix, Teklad (40060)	10.0
Ethoxyquin, antioxidant	0.04
Blue Food Color	0.15

Selected Nutrient Information

Item	*% by Weight*	*% kcal from*
Protein	17.3	15.2
Carbohydrate	48.5	42.7
Fat	21.2	42

Kcal/g = 4.5

Sodium Concentration Summary									
		**Blood**		**Heart**		**Liver**		**Kidney**	
**Generation:Sex:diet:exposure time**	**Metal Concentration (ppm)**	**Concentration**	**p value**	**Concentration**	**p value**	**Concentration**	**p value**	**Concentration**	**p value**
**F1:♂:LFD:10**	**0**	5931.3±1121(7)	0.131	906845.7±109558.12(8)	0.252	574778.6±69562.04(8)	0.131	1062638.5±86559.19(8)	0.023
**F1:♂:LFD:10**	**0.5**	5997.4±654(8)	0.871	714279.8±154054.7(8)	0.104	716173.3±99283.98(8)	0.053	937958.1±142535.47(8)	0.027
**F1:♂:LFD:10**	**5.0**	6719.8±389(7)	0.073	788521.5±358470.38(7)	0.324	678250.5±213977.49(7)	0.161	1088786±61774.94(7)	0.633
**F1:♂:LFD:24**	**0**	6459.4±1264(5)	0.088	926875.5±54015.44(4)	0.477	548021.3±88696.6(4)	0.776	1174870.6±146015.81(5)	0.011
**F1:♂:LFD:24**	**0.5**	7496.7±2038(4)	0.269	776018.4±89861.37(4)	0.809	552242.4±131211.14(4)	0.956	847241.1±43095.16(4)	0.004
**F1:♂:LFD:24**	**5.0**	5302.9±380(5)	0.196	1426565.6±1276934.93(6)	0.389	506985.6±94543.6(5)	0.574	1092932.4±165074.58(5)	0.357
**F1:♂:HFD:10**	**0**	6015.2±573(8)	0.165	857861.1±141329.42(8)	0.918	583733.8±111382.6(8)	0.214	1027146.4±115545.2(8)	0.088
**F1:♂:HFD:10**	**0.5**	6944±1615(6)	0.091	860206.5±70000.66(7)	0.968	725234.5±167106.27(7)	0.083	932100±105576.64(7)	0.083
**F1:♂:HFD:10**	**5.0**	6032.8±577(8)	0.971	838613.7±110865.53(8)	0.737	650318.3±166258.97(8)	0.384	1047683.2±77473.65(8)	0.687
**F1:♂:HFD:24**	**0**	4954.9±443(5)	0.077	929596.8±145636.77(6)	0.005	525654.4±90500.14(5)	0.465	991354.9±91612.77(6)	0.003
**F1:♂:HFD:24**	**0.5**	5684.3±594(5)	0.050	709979.8±55374.43(6)	0.003	541625.6±57780.4(6)	0.769	755095.7±79543.93(6)	0.025
**F1:♂:HFD:24**	**5.0**	4873.6±490(3)	0.834	912654.2±102701.48(6)	0.789	463412.4±131685.04(3)	0.351	1244326.5±340650.07(3)	0.045
**F1:♀:LFD:10**	**0**	7033.4±1185(6)	0.516	888830±85316.87(6)	0.28	638796.2±81819.95(6)	0.087	1096141.1±97386.1(6)	0.012
**F1:♀:LFD:10**	**0.5**	7412.9±1128(7)	0.496	844490.1±85758.06(9)	0.321	751278±142490.01(8)	0.061	860053.4±204785.45(8)	0.029
**F1:♀:LFD:10**	**5.0**	6847.7±724(10)	0.719	818541.7±78612.33(10)	0.114	648387.9±79198.06(10)	0.861	1141693.5±208680.75(10)	0.642
**F1:♀:LFD:24**	**0**	6903±2204(5)	0.747	1032544±254885.99(5)	0.145	600818.8±53230.88(4)	0.669	1240134±90479.55(5)	0.002
**F1:♀:LFD:24**	**0.5**	6195.6±2246(5)	0.529	814429.7±95183.98(5)	0.054	635024±90787.67(5)	0.699	888348.1±85502.59(5)	0.002
**F1:♀:LFD:24**	**5.0**	6185.8±646(7)	0.492	934616.7±99444.95(6)	0.339	673054.1±170492.35(7)	0.387	1240625.6±203147.3(7)	0.996
**F1:♀:HFD:10**	**0**	6711.3±1058(7)	0.558	893740.3±101744.95(7)	0.994	613659.3±102936.18(7)	0.207	1085608.6±169354.32(7)	0.723
**F1:♀:HFD:10**	**0.5**	6791.9±1654(9)	0.895	894295.3±124570.57(8)	0.993	721997.4±62532.41(8)	0.079	1151742.3±505969.74(9)	0.685
**F1:♀:HFD:10**	**5.0**	7280.5±696(10)	0.345	899393.3±122442.1(10)	0.923	672793±146841.2(10)	0.303	1212592±115148.25(10)	0.427
**F1:♀:HFD:24**	**0**	5893.2±2021(4)	0.327	968051.3±151594.36(4)	0.281	686290±86818.34(3)	0.642	1126729.8±337426.08(4)	0.016
**F1:♀:HFD:24**	**0.5**	6923.7±999(5)	0.263	831461.4±120185.35(5)	0.146	658934±90245.61(5)	0.689	868235.7±63401.31(5)	0.1
**F1:♀:HFD:24**	**5.0**	5708.4±771(5)	0.836	954560.1±38408.5(2)	0.905	585866.1±NA(1)	0.367	1345263.1±192904.69(5)	0.157
**Exposure**			NS		NS		NS		<0.001
**Diet (Low fat vs. high fat)**			NS		NS		NS		NS
**Exposure: Diet**			NS		NS		NS		NS

Sodium ANOVA Summary				
	Pr(>F)			
	**Liver**	**Kidney**	**Heart**	**Blood**
**F0**				
Cadmium Concentration	0.318	0.408	0.946	0.694
Sex	0.012	0.624	0	0.308
Cadmium Concentration:Sex	0.033	0.532	0.057	0.301
Residuals	-	-	-	-
**F1**				
Cadmium Concentration	0.004	0	0.068	0.181
Sex	0.004	0.003	0.852	0.001
Diet	0.829	0.651	0.653	0.212
Exposure Time	0	0.817	0.054	0.002
Cadmium Concentration:Sex	0.949	0.807	0.301	0.687
Cadmium Concentration:Diet	0.915	0.121	0.599	0.485
Sex:Diet	0.968	0.069	0.299	0.602
Cadmium Concentration:Exposure Time	0.094	0.004	0.064	0.093
Sex:Exposure Time	0.014	0.574	0.433	0.619
Treat:Exposure Time	0.97	0.224	0.089	0.066
Cadmium Concentration:Sex:Diet	0.93	0.473	0.383	0.681
Cadmium Concentration:Sex:Exposure Time	0.47	0.945	0.314	0.661
Cadmium Concentration:Diet:Exposure Time	0.747	0.131	0.324	0.716
Sex:Diet:Exposure Time	0.548	0.371	0.345	0.108
Cadmium Concentration:Sex:Diet:Exposure Time	0.588	0.67	0.436	0.021
Residuals	-	-	-	-

Magnesium Concentrations									
		Blood		Heart		Liver		Kidney	
**Generation:Sex:diet:exposure time**	**Metal Concentration (ppm)**	**Concentration**	**p value**	**Concentration**	**p value**	**Concentration**	**p value**	**Concentration**	**p value**
**F1:♂:LFD:10**	**0**	74.9±6.42(7)	0.017	219963.4±19822.62(8)	0.599	209802±15447.47(8)	0.064	225913.4±11568.51(8)	0.002
**F1:♂:LFD:10**	**0.5**	65.2±5.9(8)	0.005	199671.7±48253.09(8)	0.715	233573.4±23958.21(8)	0.035	201564.7±15180.2(8)	0.004
**F1:♂:LFD:10**	**5.0**	69.2±5.26(7)	0.084	257312.1±192093.57(7)	0.518	211089.9±23042.51(7)	0.907	231113±17245.82(7)	0.503
**F1:♂:LFD:24**	**0**	76.3±9.5(5)	0.233	228964.5±44128.09(4)	0.415	180871.7±10684.81(4)	0.393	253194.5±31132.94(5)	0.016
**F1:♂:LFD:24**	**0.5**	65.7±11.26(4)	0.096	202970.9±30980.04(4)	0.8	192715.1±15009.77(4)	0.182	185520.1±12771.65(4)	0.005
**F1:♂:LFD:24**	**5.0**	72.2±4.66(5)	0.466	319584.5±205749.39(6)	0.343	186446±9335.64(5)	0.493	224845.7±34310.68(5)	0.147
**F1:♂:HFD:10**	**0**	75.5±5.98(8)	0.121	233326.4±26045.47(8)	0.056	199040.2±27429.08(8)	0.569	224411±13337.6(8)	0.004
**F1:♂:HFD:10**	**0.5**	66.8±11.73(6)	0.043	202192.7±23707.47(7)	0.027	213717.9±32309.71(7)	0.339	202279.7±20437.77(7)	0.014
**F1:♂:HFD:10**	**5.0**	72.2±3.57(8)	0.383	229932.4±25731.64(8)	0.791	211545.9±27350.33(8)	0.398	233150±13846.3(8)	0.286
**F1:♂:HFD:24**	**0**	86.2±4.12(5)	0.044	252257.3±24071.54(6)	0.144	130061.6±17841.84(5)	0.707	227419±16531.4(6)	0
**F1:♂:HFD:24**	**0.5**	82.8±2.45(5)	0.321	227079.7±9802.13(6)	0.053	128956.6±15838.33(6)	0.908	179961.1±13210.28(6)	0
**F1:♂:HFD:24**	**5.0**	75.1±9.27(3)	0.015	238557.8±24861.61(6)	0.271	121090.3±5643.98(3)	0.44	246165.8±2027.09(3)	0.077
**F1:♀:LFD:10**	**0**	69.1±7.33(6)	0.146	240964.7±28958.3(6)	0.146	185891.1±17139.7(6)	0.169	192680.2±17846.91(6)	0.015
**F1:♀:LFD:10**	**0.5**	62.5±9.46(7)	0.128	217457.3±30782.23(9)	0.121	206921.3±22209.72(8)	0.077	175357±40235.48(8)	0.336
**F1:♀:LFD:10**	**5.0**	61.5±5.61(10)	0.06	212595.3±23590.53(10)	0.059	203440.5±21748.79(10)	0.119	224180.9±32177.54(10)	0.075
**F1:♀:LFD:24**	**0**	70.6±11.47(5)	0.988	231442.9±56640.2(5)	0.452	177035.5±12112.7(4)	0.043	249420.3±21452.14(5)	0.001
**F1:♀:LFD:24**	**0.5**	71.6±11.99(5)	0.883	210735.8±22828.87(5)	0.367	205249.2±9838.26(5)	0.029	180584.6±1926.77(5)	0
**F1:♀:LFD:24**	**5.0**	71.3±7.67(7)	0.909	237429.6±14436.9(6)	0.782	180441.2±22243.27(7)	0.756	231280.4±28541.86(7)	0.18
**F1:♀:HFD:10**	**0**	68.3±6.25(7)	0.283	242592±25906.32(7)	0.067	185849.7±13980.11(7)	0.314	208523.4±18044.44(7)	0.495
**F1:♀:HFD:10**	**0.5**	60.1±14.24(9)	0.127	233569±23088.19(8)	0.537	204998.5±13715.4(8)	0.138	237777±101609.65(9)	0.364
**F1:♀:HFD:10**	**5.0**	62±8.09(10)	0.227	210553±32095.25(10)	0.029	198640.9±33665.02(10)	0.292	244430.3±25492.97(10)	0.257
**F1:♀:HFD:24**	**0**	82.7±11.61(4)	0.099	248212.1±25516.01(4)	0.358	167893.7±10958.15(3)	0.91	183115.8±31397.2(4)	0.001
**F1:♀:HFD:24**	**0.5**	69.5±11.23(5)	0.055	225093.8±30874.37(5)	0.266	167479.8±8664.3(5)	0.954	183593.3±11846.43(5)	0.978
**F1:♀:HFD:24**	**5.0**	69.9±2.23(5)	0.062	256919.1±29707.79(2)	0.736	163278.3±NA(1)	0.688	255314.6±28703.47(5)	0.001
**Exposure**			<0.001		NS		0.006		<0.001
**Diet (Low fat vs. high fat)**			NS		NS		<0.001		NS
**Exposure: Diet**			NS		NS		NS		NS

Magnesium ANOVA Summary				
	Pr(>F)			
	Liver	Kidney	Heart	Blood
**F0**				
Cadmium Concentration	0.256	0.09	0.198	0.083
Sex	0.008	0.513	0.819	0.017
Cadmium Concentration:Sex	0.485	0.879	0.944	0.173
Residuals	-	-	-	-
**F1**				
Cadmium Concentration	0.006	0	0.115	0
Sex	0.892	0.482	0.541	0
Diet	0	0.167	0.982	0.092
Exposure Time	0	0.881	0.109	0
Cadmium Concentration:Sex	0.751	0.077	0.087	0.854
Cadmium Concentration:Diet	0.278	0.023	0.194	0.649
Sex:Diet	0.014	0.117	0.412	0.085
Cadmium Concentration:Exposure Time	0.685	0.011	0.522	0.726
Sex:Exposure Time	0	0.85	0.447	0.337
Treat:Exposure Time	0	0.03	0.868	0.047
Cadmium Concentration:Sex:Diet	0.804	0.206	0.44	0.311
Cadmium Concentration:Sex:Exposure Time	0.386	0.877	0.93	0.729
Cadmium Concentration:Diet:Exposure Time	0.602	0.075	0.767	0.188
Sex:Diet:Exposure Time	0.084	0.062	0.692	0.42
Cadmium Concentration:Sex:Diet:Exposure Time	0.515	0.699	0.643	0.388
Residuals	-	-	-	-

Potassium Concentration Summary									
		Blood		Heart		Liver		Kidney	
**Generation:Sex:diet:exposure time**	**Metal Concentration (ppm)**	**Concentration**	**p value**	**Concentration**	**p value**	**Concentration**	**p value**	**Concentration**	**p value**
**F1:♂:LFD:10**	**0**	4063±324.27(7)	0.106	2912263±170984.98(8)	0.681	3121171.1±271661.32(8)	0.087	3391943.2±149801.95(8)	0.005
**F1:♂:LFD:10**	**0.5**	3636.5±435.97(8)	0.045	2656232.6±618153.76(8)	0.502	3408039.5±342977.03(8)	0.079	3113441.5±285144.1(8)	0.026
**F1:♂:LFD:10**	**5.0**	3714.7±376.75(7)	0.107	2594217.6±1177585.6(7)	0.421	3061413.4±310652.09(7)	0.713	3553800.8±241718.18(7)	0.193
**F1:♂:LFD:24**	**0**	3999.6±335.43(5)	0.337	3012821±439616.76(4)	0.511	2554179.7±176589.35(4)	0.075	3609735.1±353886.37(5)	0.046
**F1:♂:LFD:24**	**0.5**	3627.9±519.97(4)	0.152	2755940.5±196959.22(4)	0.91	2812771.7±151351.62(4)	0.026	2851035.1±200064.39(4)	0.016
**F1:♂:LFD:24**	**5.0**	3874.3±203.28(5)	0.593	4912381.6±4665379.8(6)	0.371	2682393.3±93948.05(5)	0.204	3357766±528665.38(5)	0.338
**F1:♂:HFD:10**	**0**	3979.8±406.8(8)	0.079	3140411.5±282106.3(8)	0.028	2787887±403939(8)	0.626	3342739.4±156572.95(8)	0.031
**F1:♂:HFD:10**	**0.5**	3390.5±594.08(6)	0.029	2745991±276109.77(7)	0.01	2998151.1±542194.53(7)	0.365	3076803.5±326642.18(7)	0.079
**F1:♂:HFD:10**	**5.0**	3831.3±405.17(8)	0.529	3039866.4±244932.64(8)	0.461	2946246±365409.07(8)	0.478	3486675.3±323471.8(8)	0.313
**F1:♂:HFD:24**	**0**	4337.8±368.23(5)	0.977	3242706.1±305554.52(6)	0.34	1831170±186575.44(5)	0.733	3130188.5±306006.81(6)	0.004
**F1:♂:HFD:24**	**0.5**	4289.9±295.36(5)	0.854	3054500.2±156869.88(6)	0.234	1788535.1±283642.67(6)	0.762	2786211.2±204690.65(6)	0.029
**F1:♂:HFD:24**	**5.0**	4285±601.31(3)	0.861	3031984.6±299333.64(6)	0.186	1699294.7±107335.9(3)	0.442	3505383.3±90886.3(3)	0.048
**F1:♀:LFD:10**	**0**	3734.1±327.6(6)	0.099	3121812.3±383960.89(6)	0.119	2779163.4±276397.39(6)	0.063	2974957.8±315323.31(6)	0.018
**F1:♀:LFD:10**	**0.5**	3407.6±455.48(7)	0.11	2851912.6±296251.34(9)	0.093	3148383.8±316543.98(8)	0.05	2707852.3±669460.88(8)	0.358
**F1:♀:LFD:10**	**5.0**	3330±274.24(10)	0.037	2807801.1±220199.49(10)	0.049	3184602.3±364352.39(10)	0.027	3475332.3±492765.16(10)	0.08
**F1:♀:LFD:24**	**0**	3811.5±638.82(5)	0.389	2985651.3±636752.26(5)	0.371	2714057.5±163846.31(4)	0.372	3571135.5±311397.61(5)	0.001
**F1:♀:LFD:24**	**0.5**	3042.4±1667.88(5)	0.24	2799351.3±176758.48(5)	0.449	2934736.4±145291.48(5)	0.185	2704817.4±85462.04(5)	0
**F1:♀:LFD:24**	**5.0**	3778.9±406.91(7)	0.956	3133439.3±141981.04(6)	0.529	2786912.2±303412.32(7)	0.629	3344920.2±358524.11(7)	0.206
**F1:♀:HFD:10**	**0**	3604.9±283.32(7)	0.255	3067946.5±215399.63(7)	0.037	2771974.7±241455.77(7)	0.233	3130136.7±291677.15(7)	0.55
**F1:♀:HFD:10**	**0.5**	3192.6±823.93(9)	0.158	3012788.8±270746.66(8)	0.7	3101193.5±223345.6(8)	0.124	3617877.3±1588573.21(9)	0.331
**F1:♀:HFD:10**	**5.0**	3173.7±384.44(10)	0.133	2732273.8±306386.91(10)	0.02	3068989.8±556096.99(10)	0.144	3597758.6±361657.38(10)	0.341
**F1:♀:HFD:24**	**0**	3938.8±816.9(4)	0.225	3055065.7±269440.77(4)	0.63	2499678.1±234685.94(3)	0.726	2545090.3±594148.3(4)	0.016
**F1:♀:HFD:24**	**0.5**	3235±563.47(5)	0.091	2924937.8±289698.56(5)	0.504	2430092.4±79315.43(5)	0.549	2745188±186247.09(5)	0.516
**F1:♀:HFD:24**	**5.0**	3573.9±256.89(5)	0.358	3133946.9±245372.45(2)	0.751	2372757.6±NA(1)	0.492	3514545.7±494009.75(5)	0.008
**Exposure**			<0.001		NS		0.003		<0.001
**Diet (Low fat vs. high fat)**			NS		NS		<0.001		NS
**Exposure: Diet**			NS		NS		NS		NS

Potassium ANOVA Summary				
	Pr(>F)			
	**Liver**	**Kidney**	**Heart**	**Blood**
**F0**				
Cadmium Concentration	0.265	0.25	0.18	0.07
Sex	0.322	0.304	0.297	0.463
Cadmium Concentration:Sex	0.233	0.63	0.786	0.301
Residuals	-	-	-	-
**F1**				
Cadmium Concentration	0.003	0	0.374	0
Sex	0.005	0.392	0.41	0
Diet	0	0.639	0.858	0.954
Exposure Time	0	0.066	0.053	0.011
Cadmium Concentration:Sex	0.573	0.26	0.353	0.772
Cadmium Concentration:Diet	0.436	0.03	0.36	0.937
Sex:Diet	0.003	0.174	0.687	0.19
Cadmium Concentration:Exposure Time	0.437	0.162	0.143	0.512
Sex:Exposure Time	0	0.851	0.188	0.484
Treat:Exposure Time	0	0.036	0.23	0.058
Cadmium Concentration:Sex:Diet	0.694	0.214	0.639	0.747
Cadmium Concentration:Sex:Exposure Time	0.364	0.871	0.7	0.193
Cadmium Concentration:Diet:Exposure Time	0.513	0.112	0.15	0.46
Sex:Diet:Exposure Time	0.264	0.096	0.292	0.359
Cadmium Concentration:Sex:Diet:Exposure Time	0.769	0.695	0.236	0.934
Residuals	-	-	-	-

Calcium Concentration Summary									
		**Blood**		**Heart**		**Liver**		**Kidney**	
**Generation:Sex:diet:exposure time**	**Metal Concentration (ppm)**	**Concentration**	**p value**	**Concentration**	**p value**	**Concentration**	**p value**	**Concentration**	**p value**
**F1:♂:LFD:10**	**0**	106.2±44.09(7)	0.246	69857.2±29049.66(8)	0.335	42086.2±22591.11(8)	0.728	71600.9±9353.02(8)	0
**F1:♂:LFD:10**	**0.5**	88.9±8.23(8)	0.249	48416.1±28381.71(8)	0.985	37324.4±8094.76(8)	0.541	8640.2±12104.64(8)	0
**F1:♂:LFD:10**	**5.0**	113.3±21.66(7)	0.641	1656428.6±4231511.66(7)	0.201	36176±10530.22(7)	0.465	78070.6±16555.24(7)	0.341
**F1:♂:LFD:24**	**0**	113.2±18.05(5)	0.995	93247.9±45502.86(4)	0.485	17132.2±12248.18(4)	0.567	75058.3±31718.78(5)	0.015
**F1:♂:LFD:24**	**0.5**	114.2±32.27(4)	0.945	45141.4±18572.56(4)	0.259	9491.9±8252.3(4)	0.354	33715.6±12432.91(4)	0.013
**F1:♂:LFD:24**	**5.0**	114.4±11.03(5)	0.93	80374.8±75723.63(6)	0.734	16515.6±12054.97(5)	0.936	79532.6±9211.83(5)	0.742
**F1:♂:HFD:10**	**0**	101.2±17.45(8)	0.074	65091.3±26931.48(8)	0.768	37328.7±19485.53(8)	0.681	70155.9±13580.75(8)	0
**F1:♂:HFD:10**	**0.5**	86.2±22.5(6)	0.142	55365.9±22660.07(7)	0.522	31504.6±8313.26(7)	0.453	7880.7±15877.3(7)	0
**F1:♂:HFD:10**	**5.0**	110.1±14.88(8)	0.338	64723.8±34749.07(8)	0.98	37470±13335.07(8)	0.985	72009±7315.85(8)	0.772
**F1:♂:HFD:24**	**0**	126.2±25.41(5)	0.526	85858.8±28310.67(6)	0.056	18806.1±13636.6(5)	0.712	39238±38004.85(6)	0.169
**F1:♂:HFD:24**	**0.5**	108.5±20.31(5)	0.295	51196±13355.45(6)	0.02	19175.2±5064.11(6)	0.948	39920.9±28812.33(6)	0.972
**F1:♂:HFD:24**	**5.0**	123.9±32.9(3)	0.903	63529.5±24637.4(6)	0.113	14029.4±4967.62(3)	0.49	83135.9±30456.46(3)	0.086
**F1:♀:LFD:10**	**0**	94.4±13.26(6)	0.512	75769.3±25595.1(6)	0.073	38034.9±20823.97(6)	0.97	68042±23072.9(6)	0
**F1:♀:LFD:10**	**0.5**	81±19.97(7)	0.759	46655.2±17020.82(9)	0.027	40430.3±15597.17(8)	0.824	8268.3±13613.94(8)	0.001
**F1:♀:LFD:10**	**5.0**	124.2±114.1(10)	0.466	53582.7±26432.87(10)	0.078	40254±21881.79(10)	0.83	93326.9±36107.64(10)	0.088
**F1:♀:LFD:24**	**0**	111.2±23.05(5)	0.368	86818.9±66410.33(5)	0.415	14143.9±6378.53(4)	0.002	87614.3±51535.91(5)	0.065
**F1:♀:LFD:24**	**0.5**	249.5±336.19(5)	0.245	46693.5±12323.28(5)	0.25	4222.8±4659.96(5)	0.122	46089.9±20938.52(5)	0.082
**F1:♀:LFD:24**	**5.0**	107.5±7.43(7)	0.972	84733.8±59747.7(6)	0.949	28355.1±11766.62(7)	0.025	97665.7±28382.71(7)	0.632
**F1:♀:HFD:10**	**0**	97.4±12.79(7)	0.679	98792.5±61228.64(7)	0.339	48758.2±32763.77(7)	0.336	73926.4±16640.93(7)	0
**F1:♀:HFD:10**	**0.5**	89.2±41.03(9)	0.562	89956.2±37496.18(8)	0.699	46369.6±18317.13(8)	0.842	22131.9±37297.11(9)	0.001
**F1:♀:HFD:10**	**5.0**	85.4±18.57(10)	0.387	68142.2±32567.17(10)	0.168	33491±17293.76(10)	0.189	80972±16080.46(10)	0.582
**F1:♀:HFD:24**	**0**	126.4±18.55(4)	0.354	113275.8±61324.78(4)	0.33	13225.2±4046.31(3)	0.311	43606.5±38940.51(4)	0.004
**F1:♀:HFD:24**	**0.5**	109.5±22.7(5)	0.224	58741.8±32205.92(5)	0.153	16902.2±5549.01(5)	0.361	26341.1±25541.55(5)	0.409
**F1:♀:HFD:24**	**5.0**	108.4±16.42(5)	0.197	92741.5±75850.24(2)	0.657	22942.2±NA(1)	0.15	106695.4±26187.94(5)	0.009
**Exposure**			NS		NS		NS		<0.001
**Diet (Low fat vs. high fat)**			NS		NS		NS		NS
**Exposure: Diet**			NS		NS		NS		NS

Calcium ANOVA Summary				
	Pr(>F)			
	**Liver**	**Kidney**	**Heart**	**Blood**
**F0**				
Cadmium Concentration	0.734	0.877	0.478	0.377
Sex	0.798	0.531	0.115	0.695
Cadmium Concentration:Sex	0.102	0.92	0.086	0.769
Residuals	-	-	-	-
**F1**				
Cadmium Concentration	0.47	0	0.414	0.986
Sex	0.202	0.044	0.347	0.746
Diet	0.505	0.181	0.354	0.258
Exposure Time	0	0.034	0.449	0.026
Cadmium Concentration:Sex	0.922	0.43	0.335	0.407
Cadmium Concentration:Diet	0.271	0.203	0.32	0.47
Sex:Diet	0.662	0.896	0.302	0.31
Cadmium Concentration:Exposure Time	0.262	0.004	0.377	0.127
Sex:Exposure Time	0.552	0.69	0.456	0.369
Treat:Exposure Time	0.593	0.139	0.465	0.647
Cadmium Concentration:Sex:Diet	0.296	0.99	0.372	0.681
Cadmium Concentration:Sex:Exposure Time	0.212	0.602	0.426	0.412
Cadmium Concentration:Diet:Exposure Time	0.574	0.019	0.366	0.129
Sex:Diet:Exposure Time	0.48	0.374	0.41	0.397
Cadmium Concentration:Sex:Diet:Exposure Time	0.759	0.442	0.501	0.298
Residuals	-	-	-	-

Manganese Concentration Summary									
		Blood		Heart		Liver		Kidney	
Generation:Sex:diet:exposure time	Metal Concentration (ppm)	Concentration	p value	Concentration	p value	Concentration	p value	Concentration	p value
**F1:♂:LFD:10**	**0**	0.1±0.02(7)	0.251	594.5±86.78(8)	0.343	721.7±116.42(8)	0.596	1504.5±206.06(8)	0.359
**F1:♂:LFD:10**	**0.5**	0±0.01(8)	0.124	622.3±295.9(8)	0.994	781.1±87.96(8)	0.316	1508.6±198.05(8)	0.971
**F1:♂:LFD:10**	**5.0**	0.1±0.02(7)	0.77	5790±13885.75(7)	0.202	748±139.9(7)	0.664	1655.3±267.06(7)	0.207
**F1:♂:LFD:24**	**0**	0±0.01(5)	0.371	725.5±167.97(4)	0.387	546.8±140.15(4)	0.684	1629.5±154.99(5)	0.056
**F1:♂:LFD:24**	**0.5**	0±0.02(4)	0.439	470.4±116.82(4)	0.901	543.9±107.94(4)	0.971	1177.4±95.28(4)	0.025
**F1:♂:LFD:24**	**5.0**	0±0.02(5)	0.492	2799.9±4212.03(6)	0.282	602.1±89.61(5)	0.48	1559.3±393.26(5)	0.678
**F1:♂:HFD:10**	**0**	0.1±0.02(8)	0.115	614.1±153.62(8)	0.639	655.4±102.46(8)	0.116	1766±348.71(8)	0.843
**F1:♂:HFD:10**	**0.5**	0.1±0.02(6)	0.994	548.2±101(7)	0.375	883.3±273.26(7)	0.042	1668.8±345.96(7)	0.588
**F1:♂:HFD:10**	**5.0**	0.1±0.02(8)	0.065	602±155.05(8)	0.865	787.3±206.31(8)	0.207	1689.9±328.89(8)	0.66
**F1:♂:HFD:24**	**0**	0.1±0.01(5)	0.89	742.8±202.49(6)	0.253	478.9±104.84(5)	0.708	1451.3±170.92(6)	0.01
**F1:♂:HFD:24**	**0.5**	0.1±0.04(5)	0.706	655.5±59.22(6)	0.43	431±110.89(6)	0.441	1252.4±199.04(6)	0.065
**F1:♂:HFD:24**	**5.0**	0.1±0.01(3)	0.674	842.3±244.68(6)	0.37	435.3±31.8(3)	0.558	1690.7±17.67(3)	0.069
**F1:♀:LFD:10**	**0**	0.1±0.01(6)	0.435	623.6±64.34(6)	0.787	584.1±111.45(6)	0	1096±71.27(6)	0.062
**F1:♀:LFD:10**	**0.5**	0.1±0.01(7)	0.901	597.3±107.53(9)	0.585	934.2±188.9(8)	0	1167.1±244.79(8)	0.624
**F1:♀:LFD:10**	**5.0**	0.1±0.11(10)	0.267	623.3±85.27(10)	0.995	671.5±117.85(10)	0.254	1407.9±337.92(10)	0.033
**F1:♀:LFD:24**	**0**	0±0.01(5)	0.677	731.7±220.42(5)	0.254	581.7±82.68(4)	0.067	1395.5±102.6(5)	0.007
**F1:♀:LFD:24**	**0.5**	0.1±0.09(5)	0.425	516.4±129.1(5)	0.289	736.5±85.24(5)	0.031	1008.7±71.4(5)	0.002
**F1:♀:LFD:24**	**5.0**	0.1±0.04(7)	0.469	838.5±440.16(6)	0.576	624±106.81(7)	0.491	1269.7±228.17(7)	0.21
**F1:♀:HFD:10**	**0**	0.1±0.01(7)	0.045	823±260.82(7)	0.038	714.2±87.42(7)	0.016	1215.3±157.17(7)	0.274
**F1:♀:HFD:10**	**0.5**	0.1±0.01(9)	0.423	685.9±214.59(8)	0.194	1105.3±286.73(8)	0.004	1515.1±638.47(9)	0.168
**F1:♀:HFD:10**	**5.0**	0.1±0.02(10)	0.018	555.5±120.11(10)	0.012	926.8±264.5(10)	0.084	1526.4±259.45(10)	0.144
**F1:♀:HFD:24**	**0**	0.1±0.03(4)	0.83	798.1±300.46(4)	0.067	806.3±196.89(3)	0.969	1151±530.5(4)	0.44
**F1:♀:HFD:24**	**0.5**	0.1±0.02(5)	0.688	642.1±185.99(5)	0.37	839.6±246.44(5)	0.85	1090.3±214.75(5)	0.813
**F1:♀:HFD:24**	**5.0**	0.1±0.01(5)	0.882	1210.4±265.37(2)	0.088	865.9±NA(1)	0.83	1389.6±355.04(5)	0.361
**Exposure**			NS		NS		<0.001		0.011
**Diet (Low fat vs. high fat)**			NS		NS		<0.001		0.033
**Exposure: Diet**			NS		NS		NS		NS

Manganese ANOVA Summary				
	Pr(>F)			
	**Liver**	**Kidney**	**Heart**	**Blood**
**F0**				
Cadmium Concentration	0.435	0.494	0.684	0.854
Sex	0.732	0.001	0.256	0.614
Cadmium Concentration:Sex	0.674	0.643	0.911	0.692
Residuals	-	-	-	-
**F1**				
Cadmium Concentration	0	0.011	0.205	0.09
Sex	0	0	0.227	0.222
Diet	0.004	0.033	0.241	0.353
Exposure Time	0	0.003	0.863	0.52
Cadmium Concentration:Sex	0.006	0.298	0.188	0.458
Cadmium Concentration:Diet	0.54	0.426	0.173	0.941
Sex:Diet	0.001	0.747	0.187	0.166
Cadmium Concentration:Exposure Time	0.012	0.018	0.782	0.286
Sex:Exposure Time	0.006	0.444	0.72	0.617
Treat:Exposure Time	0.18	0.07	0.659	0.154
Cadmium Concentration:Sex:Diet	0.548	0.582	0.22	0.344
Cadmium Concentration:Sex:Exposure Time	0.811	0.47	0.704	0.935
Cadmium Concentration:Diet:Exposure Time	0.388	0.192	0.658	0.642
Sex:Diet:Exposure Time	0.254	0.732	0.633	0.764
Cadmium Concentration:Sex:Diet:Exposure Time	0.956	0.868	0.847	0.895
Residuals	-	-	-	-

Iron Concentration Summary									
		**Blood**		**Heart**		**Liver**		**Kidney**	
**Generation:Sex:diet:exposure time**	**Metal Concentration (ppm)**	**Concentration**	**p value**	**Concentration**	**p value**	**Concentration**	**p value**	**Concentration**	**p value**
**F1:♂:LFD:10**	**0**	870.1±58.22(7)	0.09	86583.9±11962.53(8)	0.437	63471.3±11591.34(8)	0.323	64101.4±8199.54(8)	0.026
**F1:♂:LFD:10**	**0.5**	780.7±95.83(8)	0.047	73853.9±20993.7(8)	0.843	66457.3±11753.03(8)	0.588	57611.4±7343.4(8)	0.085
**F1:♂:LFD:10**	**5.0**	785.3±83.07(7)	0.066	155010±230551.5(7)	0.311	57847.4±8664.64(7)	0.329	68449.7±5416.69(7)	0.254
**F1:♂:LFD:24**	**0**	859.7±58.07(5)	0.478	101101.1±3886.6(4)	0.072	82305±18465.3(4)	0.737	111242.1±44022.3(5)	0.057
**F1:♂:LFD:24**	**0.5**	800.3±108(4)	0.237	84114.2±4995.71(4)	0.584	74829±36666.15(4)	0.742	64803.5±9372.47(4)	0.031
**F1:♂:LFD:24**	**5.0**	828.7±40.19(5)	0.503	150911.3±63040.3(6)	0.098	65773.5±34371.6(5)	0.449	72316±12076.26(5)	0.05
**F1:♂:HFD:10**	**0**	848.2±85.13(8)	0.063	78908.9±9956.63(8)	0.692	43558.8±8081.41(8)	0.921	61401.6±5087.63(8)	0.39
**F1:♂:HFD:10**	**0.5**	711.3±122.8(6)	0.022	80104.5±10211.62(7)	0.821	45779.9±16301.69(7)	0.727	55257.3±12191.83(7)	0.175
**F1:♂:HFD:10**	**5.0**	809.1±99.59(8)	0.45	75789.2±10010.85(8)	0.542	43524.7±11241.48(8)	0.996	58573.9±7111.95(8)	0.511
**F1:♂:HFD:24**	**0**	885.8±80.8(5)	0.949	94117.3±9650.27(6)	0.454	23763.9±4710.14(5)	0.53	59925.4±8809.61(6)	0.074
**F1:♂:HFD:24**	**0.5**	879.3±73.38(5)	0.908	86247.4±9074.8(6)	0.555	27833.3±6730.3(6)	0.274	48306.5±7429.08(6)	0.028
**F1:♂:HFD:24**	**5.0**	899.7±115.95(3)	0.83	103064.4±36813.74(6)	0.503	25484.5±5436.87(3)	0.694	56793.1±7472.76(3)	0.592
**F1:♀:LFD:10**	**0**	807.2±67.61(6)	0.093	94864.1±14979.42(6)	0.021	93082.8±8417.92(6)	0.69	68905.2±6453.28(6)	0.931
**F1:♀:LFD:10**	**0.5**	740.3±99.4(7)	0.13	86916.2±7719.52(9)	0.131	100395.9±28958.52(8)	0.507	64755.6±30125.39(8)	0.727
**F1:♀:LFD:10**	**5.0**	717.1±61.11(10)	0.033	79858.7±6961.59(10)	0.006	92717.5±15753.27(10)	0.972	67717.5±19168.92(10)	0.917
**F1:♀:LFD:24**	**0**	843.3±141.83(5)	0.42	93162.4±16741.61(5)	0.135	135795.9±6718.39(4)	0.035	117477.2±15520.37(5)	0.08
**F1:♀:LFD:24**	**0.5**	668.4±364.78(5)	0.223	97830.1±13386.6(5)	0.761	198055.7±5866.15(5)	0.035	89774.6±14520.49(5)	0.049
**F1:♀:LFD:24**	**5.0**	806±85.93(7)	0.773	121751.7±33110.21(6)	0.068	134268.3±57803.37(7)	0.952	91545±25796.6(7)	0.047
**F1:♀:HFD:10**	**0**	775.4±54.91(7)	0.21	90133.2±15035.26(7)	0.41	56892.4±5356.72(7)	0.735	53898±6146.06(7)	0.413
**F1:♀:HFD:10**	**0.5**	688.7±175.75(9)	0.16	86923.9±14719.44(8)	0.65	55678.4±15258.26(8)	0.868	68632.8±36181.77(9)	0.208
**F1:♀:HFD:10**	**5.0**	673.7±79.52(10)	0.094	81361.6±11197.67(10)	0.2	51854.1±16541.93(10)	0.471	58563.2±10564.99(10)	0.679
**F1:♀:HFD:24**	**0**	799.2±209.11(4)	0.41	86509.1±26507.94(4)	0.425	34828.8±20282.91(3)	0.838	51165.4±19056.7(4)	0.23
**F1:♀:HFD:24**	**0.5**	671.9±119.1(5)	0.194	75286.1±10219.44(5)	0.453	36055.9±9875.47(5)	0.91	42061.8±4263.58(5)	0.423
**F1:♀:HFD:24**	**5.0**	739.2±68.93(5)	0.528	99084.3±32810.93(2)	0.513	26662.9±NA(1)	0.637	60991.9±21010.75(5)	0.388
**Exposure**			<0.001		NS		NS		0.019
**Diet (Low fat vs. high fat)**			NS		NS		<0.001		<0.001
**Exposure: Diet**			NS		NS		0.016		NS

Iron ANOVA Summary				
	Pr(>F)			
	Liver	Kidney	Heart	Blood
**F0**				
Cadmium Concentration	0.08	0.93	0.911	0.106
Sex	0.113	0	0.502	0.608
Cadmium Concentration:Sex	0.656	0.019	0.248	0.452
Residuals	-	-	-	-
**F1**				
Cadmium Concentration	0.111	0.019	0.112	0
Sex	0	0.06	0.423	0
Diet	0	0	0.074	0.325
Exposure Time	0.001	0.001	0.153	0.047
Cadmium Concentration:Sex	0.324	0.336	0.196	0.759
Cadmium Concentration:Diet	0.016	0.046	0.187	0.999
Sex:Diet	0	0.117	0.342	0.194
Cadmium Concentration:Exposure Time	0.275	0.006	0.749	0.469
Sex:Exposure Time	0	0.427	0.835	0.406
Treat:Exposure Time	0	0	0.723	0.325
Cadmium Concentration:Sex:Diet	0.13	0.849	0.205	0.688
Cadmium Concentration:Sex:Exposure Time	0.152	0.53	0.642	0.196
Cadmium Concentration:Diet:Exposure Time	0.185	0.061	0.878	0.585
Sex:Diet:Exposure Time	0	0.117	0.491	0.351
Cadmium Concentration:Sex:Diet:Exposure Time	0.145	0.489	0.835	0.985
Residuals	-	-	-	-

Molybdenum Concentration Summary									
		Blood		Heart		Liver		Kidney	
**Generation:Sex:diet:exposure time**	**Metal Concentration (ppm)**	**Concentration**	**p value**	**Concentration**	**p value**	**Concentration**	**p value**	**Concentration**	**p value**
**F1:♂:LFD:10**	**0**	0±0.01(7)	0.511	38.4±10.5(8)	0.342	718±109.46(8)	0.215	363.7±26.29(8)	0.231
**F1:♂:LFD:10**	**0.5**	0±0(8)	0.344	32.3±7.99(8)	0.907	777.9±83.98(8)	0.223	369.8±20.33(8)	0.646
**F1:♂:LFD:10**	**5.0**	0±0(7)	0.942	104.3±184.6(7)	0.223	691.1±89.33(7)	0.59	386.9±30.95(7)	0.1
**F1:♂:LFD:24**	**0**	0±0(5)	0.773	35.8±3.54(4)	0.508	624.2±99.4(4)	0.249	404.6±72.04(5)	0.239
**F1:♂:LFD:24**	**0.5**	0±0(4)	0.497	32±3.31(4)	0.988	683.5±79.16(4)	0.275	328.3±31.39(4)	0.098
**F1:♂:LFD:24**	**5.0**	0±0(5)	0.871	261.3±519.9(6)	0.34	597.1±33.25(5)	0.591	371.1±70.41(5)	0.418
**F1:♂:HFD:10**	**0**	0±0(8)	0.608	40.9±7.57(8)	0.362	746.9±94.09(8)	0.137	371.4±33.84(8)	0.196
**F1:♂:HFD:10**	**0.5**	0±0(6)	0.926	34.7±9.78(7)	0.16	863.4±119.48(7)	0.049	381±53.15(7)	0.62
**F1:♂:HFD:10**	**5.0**	0±0(8)	0.361	38.4±7.68(8)	0.551	801±108.67(8)	0.325	405.2±18.41(8)	0.082
**F1:♂:HFD:24**	**0**	0±0(5)	0.124	43.1±5.81(6)	0.101	399.6±163.78(5)	0.979	437.8±26.93(6)	0
**F1:♂:HFD:24**	**0.5**	0±0(5)	0.855	37.7±8.37(6)	0.724	407.9±66.92(6)	0.903	357.5±39.4(6)	0.001
**F1:♂:HFD:24**	**5.0**	0±0(3)	0.078	70±43.76(6)	0.092	415.7±26.19(3)	0.844	474.7±17.72(3)	0.126
**F1:♀:LFD:10**	**0**	0±0(6)	0.572	49.5±8.23(6)	0.037	636.6±89.93(6)	0.175	295.8±41.33(6)	0.046
**F1:♀:LFD:10**	**0.5**	0±0(7)	0.345	36.4±11.83(9)	0.036	711.2±59.6(8)	0.092	292.1±60(8)	0.905
**F1:♀:LFD:10**	**5.0**	0±0(10)	0.359	34.1±11.83(10)	0.014	652.8±83.55(10)	0.694	356±60.68(10)	0.051
**F1:♀:LFD:24**	**0**	0±0(5)	0.049	41±12.24(5)	0.036	591.7±33.82(4)	0.008	311.4±27.02(5)	0.051
**F1:♀:LFD:24**	**0.5**	0±0(5)	0.301	23.9±7.89(5)	0.209	718.4±42.69(5)	0.014	285.5±28.15(5)	0.196
**F1:♀:LFD:24**	**5.0**	0±0(7)	0.017	60.2±30.2(6)	0.143	578.2±87.87(7)	0.749	333.5±33.21(7)	0.23
**F1:♀:HFD:10**	**0**	0±0(7)	0.172	46.2±16.35(7)	0.234	667.3±92.95(7)	0.252	315.4±20.94(7)	0.294
**F1:♀:HFD:10**	**0.5**	0±0(9)	0.357	45.5±13.63(8)	0.921	758.8±101.33(8)	0.157	372.1±132.17(9)	0.198
**F1:♀:HFD:10**	**5.0**	0±0(10)	0.4	35.9±11.83(10)	0.144	759.9±147.86(10)	0.134	378.4±51.21(10)	0.146
**F1:♀:HFD:24**	**0**	0±0(4)	0.239	61.2±19.56(4)	0.335	611.1±106.78(3)	0.971	309±65.8(4)	0.011
**F1:♀:HFD:24**	**0.5**	0±0(5)	0.151	42.2±18.86(5)	0.155	595.5±75.9(5)	0.815	319.8±18.01(5)	0.776
**F1:♀:HFD:24**	**5.0**	0±0(5)	0.129	53.9±4.47(2)	0.653	602.7±NA(1)	0.937	430±69.05(5)	0.007
**Exposure**			NS		NS		0.013		<0.001
**Diet (Low fat vs. high fat)**			NS		NS		NS		<0.001
**Exposure: Diet**			NS		NS		NS		NS

Molybdenum ANOVA Summary				
	Pr(>F)			
	**Liver**	**Kidney**	**Heart**	**Blood**
**F0**				
Cadmium Concentration	0.484	0.092	0.759	0.605
Sex	0	0.022	0.043	0.017
Cadmium Concentration:Sex	0.049	0.753	0.153	0.101
Residuals	-	-	-	-
**F1**				
Cadmium Concentration	0.013	0	0.138	0.134
Sex	0.87	0	0.236	0.615
Diet	0.892	0	0.326	0.594
Exposure Time	0	0.618	0.199	0
Cadmium Concentration:Sex	0.85	0.047	0.139	0.477
Cadmium Concentration:Diet	0.047	0.305	0.19	0.035
Sex:Diet	0.061	0.367	0.172	0.918
Cadmium Concentration:Exposure Time	0.697	0.01	0.339	0.541
Sex:Exposure Time	0	0.326	0.409	0.127
Treat:Exposure Time	0	0.196	0.543	0.885
Cadmium Concentration:Sex:Diet	0.847	0.405	0.296	0.585
Cadmium Concentration:Sex:Exposure Time	0.301	0.485	0.682	0.481
Cadmium Concentration:Diet:Exposure Time	0.37	0.076	0.55	0.516
Sex:Diet:Exposure Time	0.003	0.254	0.545	0.44
Cadmium Concentration:Sex:Diet:Exposure Time	0.952	0.826	0.807	0.599
Residuals	-	-	-	-

Selenium Concentration Summary									
		Blood		Heart		Liver		Kidney	
Generation:Sex:diet:exposure time	Metal Concentration (ppm)	Concentration	p value	Concentration	p value	Concentration	p value	Concentration	p value
**F1:♂:LFD:10**	**0**	1.3±0.36(7)	0.912	177.9±503.04(8)	0.763	1085.1±652.94(8)	0.402	1826.7±937.8(8)	0.003
**F1:♂:LFD:10**	**0.5**	1.2±0.18(8)	0.682	64.4±119.88(8)	0.497	1196.9±806.16(8)	0.742	172.7±394.78(8)	0.001
**F1:♂:LFD:10**	**5.0**	1.2±0.26(7)	0.762	81.8±216.54(7)	0.578	734.1±487.5(7)	0.323	1630.5±1206.2(7)	0.676
**F1:♂:LFD:24**	**0**	0.4±0.22(5)	0.763	0±0(4)	0.392	609±361.52(4)	0.959	1818.3±633.13(5)	0.206
**F1:♂:LFD:24**	**0.5**	0.5±0.24(4)	0.941	0±0(4)	1	550±264.12(4)	0.828	496.1±992.18(4)	0.113
**F1:♂:LFD:24**	**5.0**	0.4±0.24(5)	0.549	134±257.48(6)	0.257	618.4±444.5(5)	0.971	1763.8±1569.23(5)	0.941
**F1:♂:HFD:10**	**0**	1.1±0.54(8)	0.965	104.7±280.79(8)	0.756	773±598.05(8)	0.628	1336±852.55(8)	0.081
**F1:♂:HFD:10**	**0.5**	1.1±0.43(6)	0.887	25.1±66.53(7)	0.578	803.5±613.67(7)	0.928	0±0(7)	0.048
**F1:♂:HFD:10**	**5.0**	1.1±0.37(8)	0.794	126.9±358.93(8)	0.872	1060.4±704.85(8)	0.381	1325.4±1892.95(8)	0.986
**F1:♂:HFD:24**	**0**	0.5±0.38(5)	0.522	0±0(6)	0.391	447.2±420.62(5)	0.466	1252.4±1135.16(6)	0.005
**F1:♂:HFD:24**	**0.5**	0.6±0.4(5)	0.502	44.6±109.26(6)	0.24	357.1±111.12(6)	0.604	0±0(6)	0.016
**F1:♂:HFD:24**	**5.0**	0.3±0.08(3)	0.591	0±0(6)	1	186.7±207.54(3)	0.227	2112.8±591.74(3)	0.141
**F1:♀:LFD:10**	**0**	0.9±0.67(6)	0.465	624.2±1528.9(6)	0.219	524.7±375.62(6)	0.216	1059.4±1071.13(6)	0.027
**F1:♀:LFD:10**	**0.5**	1.2±0.57(7)	0.301	17.3±51.83(9)	0.129	1080.7±675.52(8)	0.089	256.2±724.69(8)	0.263
**F1:♀:LFD:10**	**5.0**	1.2±0.24(10)	0.256	0±0(10)	0.112	928.8±588.37(10)	0.19	2047.9±1687.73(10)	0.153
**F1:♀:LFD:24**	**0**	0.3±0.3(5)	0.671	0±0(5)	NaN	483.2±454.1(4)	0.801	2393.8±1720.77(5)	0.086
**F1:♀:LFD:24**	**0.5**	0.6±0.62(5)	0.394	0±0(5)	NaN	403.3±363.7(5)	0.737	657.6±1470.55(5)	0.063
**F1:♀:LFD:24**	**5.0**	0.5±0.38(7)	0.523	0±0(6)	NaN	337.9±263.13(7)	0.515	2422.8±941.17(7)	0.971
**F1:♀:HFD:10**	**0**	1±0.73(7)	0.666	141.9±375.43(7)	0.287	719.7±453.72(7)	0.096	1929.6±1262.25(7)	0.047
**F1:♀:HFD:10**	**0.5**	1±0.36(9)	0.746	0±0(8)	0.176	1439.1±674.75(8)	0.039	464.8±1272.2(9)	0.052
**F1:♀:HFD:10**	**5.0**	1.2±0.29(10)	0.386	0±0(10)	0.156	939.3±697.79(10)	0.489	2064.8±1624.91(10)	0.848
**F1:♀:HFD:24**	**0**	0.4±0.11(4)	0.16	0±0(4)	NaN	230.6±167.46(3)	0.308	3344.2±1991.49(4)	0.006
**F1:♀:HFD:24**	**0.5**	0.2±0.27(5)	0.46	0±0(5)	NaN	368.5±245.67(5)	0.429	136.8±305.78(5)	0.002
**F1:♀:HFD:24**	**5.0**	0.6±0.45(5)	0.26	0±0(2)	NaN	664.4±NA(1)	0.143	938.4±967.17(5)	0.013
**Exposure**			NS		NS		NS		<0.001
**Diet (Low fat vs. high fat)**			NS		NS		NS		NS
**Exposure: Diet**			NS		NS		NS		NS

Selenium ANOVA Summary				
	Pr(>F)			
	**Liver**	**Kidney**	**Heart**	**Blood**
**F0**				
Cadmium Concentration	0.395	0.001	-	0.705
Sex	0.948	0.971	-	0.594
Cadmium Concentration:Sex	0.499	0.976	-	0.89
Residuals	-	-	-	-
**F1**				
Cadmium Concentration	0.282	0	0.182	0.28
Sex	0.866	0.065	0.96	0.383
Diet	0.956	0.397	0.355	0.698
Exposure Time	0	0.311	0.16	0
Cadmium Concentration:Sex	0.242	0.87	0.237	0.255
Cadmium Concentration:Diet	0.694	0.694	0.568	0.782
Sex:Diet	0.149	0.341	0.589	0.825
Cadmium Concentration:Exposure Time	0.247	0.463	0.212	0.915
Sex:Exposure Time	0.679	0.618	0.577	0.822
Treat:Exposure Time	0.43	0.294	0.649	0.435
Cadmium Concentration:Sex:Diet	0.461	0.192	0.58	0.435
Cadmium Concentration:Sex:Exposure Time	0.595	0.092	0.541	0.597
Cadmium Concentration:Diet:Exposure Time	0.86	0.792	0.465	0.935
Sex:Diet:Exposure Time	0.669	0.318	0.492	0.512
Cadmium Concentration:Sex:Diet:Exposure Time	0.204	0.478	0.722	0.681
Residuals	-	-	-	-

Zinc Concentration Summary										
		Blood		Heart		Liver		Kidney	
**Generation:Sex:diet:exposure time**	**Metal Concentration (ppm)**	**Concentration**	**p value**	**Concentration**	**p value**	**Concentration**	**p value**	**Concentration**	**p value**
**F1:♂:LFD:10**	**0**	7.5±0.6(7)	0.168	16760±2250.16(8)	0.697	23426.8±3528.73(8)	0.264	22498.1±968.03(8)	0.002
**F1:♂:LFD:10**	**0.5**	6.7±1.02(8)	0.078	14624.3±4386.22(8)	0.414	26180±3283.77(8)	0.128	20986.8±1650.49(8)	0.042
**F1:♂:LFD:10**	**5.0**	6.8±0.84(7)	0.139	15235.1±7682.02(7)	0.571	23910.6±3588.16(7)	0.79	24041.6±1485.31(7)	0.045
**F1:♂:LFD:24**	**0**	7.9±0.66(5)	0.384	15855.1±1722.57(4)	0.587	19983.1±3586.42(4)	0.912	27390.5±5550.78(5)	0.049
**F1:♂:LFD:24**	**0.5**	7.4±0.55(4)	0.223	19144.5±6688.32(4)	0.797	20085.7±1143.8(4)	0.949	19474±1872.72(4)	0.016
**F1:♂:LFD:24**	**5.0**	7.5±0.23(5)	0.262	27266.2±25631.12(6)	0.338	19499.5±1197.77(5)	0.749	24380±3791.13(5)	0.278
**F1:♂:HFD:10**	**0**	7.4±0.99(8)	0.129	17655.4±1818.11(8)	0.033	23845.6±3010.6(8)	0.356	23041.6±1547.74(8)	0.02
**F1:♂:HFD:10**	**0.5**	6.4±1.31(6)	0.055	15410.6±1900.79(7)	0.026	26208.1±4038.93(7)	0.303	21292.1±2497.41(7)	0.125
**F1:♂:HFD:10**	**5.0**	7.3±0.51(8)	0.704	15412.3±1691.38(8)	0.022	26892.2±5490.28(8)	0.173	24681.1±2231.75(8)	0.136
**F1:♂:HFD:24**	**0**	8.7±0.75(5)	0.647	17204.9±3427.63(6)	0.538	14425.6±3434.91(5)	0.908	21211.4±1652.7(6)	0.41
**F1:♂:HFD:24**	**0.5**	8.3±0.38(5)	0.397	15617.7±1783.08(6)	0.351	14185.6±1705.26(6)	0.873	22086.3±7422.2(6)	0.764
**F1:♂:HFD:24**	**5.0**	8.4±0.96(3)	0.497	15551.3±3080.12(6)	0.331	13648.4±1187.08(3)	0.669	25952.4±1081.27(3)	0.199
**F1:♀:LFD:10**	**0**	7.1±0.7(6)	0.015	17080.1±3725.67(6)	0.437	22570.1±3360.72(6)	0.126	19340.6±1539.86(6)	0.001
**F1:♀:LFD:10**	**0.5**	6.3±0.89(7)	0.03	15842.3±2757.27(9)	0.428	26111.6±2586.47(8)	0.052	17148.9±4390.93(8)	0.346
**F1:♀:LFD:10**	**5.0**	6±0.38(10)	0.005	15110±2499.2(10)	0.204	25336.4±3474.57(10)	0.107	25617.7±5000.84(10)	0.009
**F1:♀:LFD:24**	**0**	7.9±1.22(5)	0.436	15218.7±3657.15(5)	0.557	20015±878.66(4)	0.002	22237.5±2742.19(5)	0.004
**F1:♀:LFD:24**	**0.5**	6.9±1.61(5)	0.208	13562±4569.4(5)	0.454	26021.8±2239.18(5)	0.001	19758.2±2105.37(5)	0.114
**F1:♀:LFD:24**	**5.0**	7.5±0.76(7)	0.557	15778.8±1581.93(6)	0.789	22704.4±2194.58(7)	0.05	25286.2±2164.99(7)	0.042
**F1:♀:HFD:10**	**0**	7±0.9(7)	0.193	15837.1±1445.86(7)	0.802	23787±2247.02(7)	0.17	20572.9±1283.92(7)	0.096
**F1:♀:HFD:10**	**0.5**	6.1±1.64(9)	0.126	14499.9±2716.01(8)	0.698	27129.1±2719.52(8)	0.115	22802.1±8663.43(9)	0.45
**F1:♀:HFD:10**	**5.0**	6±0.91(10)	0.096	16574.7±9934.22(10)	0.822	27296.8±5358.85(10)	0.084	26809.2±4123.28(10)	0.038
**F1:♀:HFD:24**	**0**	8±1.23(4)	0.31	17546.2±1561.29(4)	0.286	20709.8±1363.81(3)	0.272	17147.7±3144.3(4)	0
**F1:♀:HFD:24**	**0.5**	7±0.99(5)	0.134	14740.4±3299.41(5)	0.145	20768.3±798.85(5)	0.94	18847.6±1434.73(5)	0.414
**F1:♀:HFD:24**	**5.0**	7.4±0.57(5)	0.371	14808.1±1709.65(2)	0.257	22702.3±NA(1)	0.142	30803.1±3871.35(5)	0
**Exposure**			<0.001		NS		0.001		<0.001
**Diet (Low fat vs. high fat)**			NS		NS		NS		NS
**Exposure: Diet**			NS		NS		NS		NS

Zinc ANOVA Summary				
	Pr(>F)			
	**Liver**	**Kidney**	**Heart**	**Blood**
**F0**				
Cadmium Concentration	0.033	0	0.561	0.168
Sex	0.142	0.098	0.436	0.323
Cadmium Concentration:Sex	0.008	0.019	0.095	0.275
Residuals	-	-	-	-
**F1**				
Cadmium Concentration	0.001	0	0.394	0
Sex	0	0.246	0.199	0
Diet	0.517	0.158	0.487	0.481
Exposure Time	0	0.447	0.285	0
Cadmium Concentration:Sex	0.326	0.002	0.829	0.547
Cadmium Concentration:Diet	0.134	0.016	0.579	0.912
Sex:Diet	0.205	0.193	0.295	0.156
Cadmium Concentration:Exposure Time	0.685	0.498	0.442	0.664
Sex:Exposure Time	0	0.78	0.14	0.919
Treat:Exposure Time	0	0.145	0.265	0.136
Cadmium Concentration:Sex:Diet	0.74	0.932	0.373	0.803
Cadmium Concentration:Sex:Exposure Time	0.658	0.726	0.452	0.341
Cadmium Concentration:Diet:Exposure Time	0.594	0.011	0.172	0.747
Sex:Diet:Exposure Time	0.157	0.494	0.147	0.266
Cadmium Concentration:Sex:Diet:Exposure Time	0.424	0.125	0.845	0.953
Residuals	-	-	-	-

Copper Concentration Summary									
		Blood		Heart		Liver		Kidney	
**Generation:Sex:diet:exposure time**	**Metal Concentration (ppm)**	**Concentration**	**p value**	**Concentration**	**p value**	**Concentration**	**p value**	**Concentration**	**p value**
**F1:♂:LFD:10**	**0**	1.1±0.19(7)	0.024	6494.7±734.08(8)	0.872	4500.5±524.28(8)	0.261	5473.9±1104.16(8)	0.064
**F1:♂:LFD:10**	**0.5**	0.9±0.1(8)	0.019	6015.1±1422.25(8)	0.608	4462.8±667.55(8)	0.891	4737.9±413.88(8)	0.054
**F1:♂:LFD:10**	**5.0**	1.1±0.15(7)	0.962	6204.6±2879.92(7)	0.764	4899.6±389.12(7)	0.173	5577.2±306.64(7)	0.784
**F1:♂:LFD:24**	**0**	1.1±0.12(5)	0.342	6203.3±1313.31(4)	0.63	3589.2±286.3(4)	0.232	5322.2±613.09(5)	0.017
**F1:♂:LFD:24**	**0.5**	1.1±0.14(4)	0.976	7649.3±4364.8(4)	0.75	3473.4±297.7(4)	0.58	3919.7±241.74(4)	0.005
**F1:♂:LFD:24**	**5.0**	1±0.07(5)	0.197	10051.6±8574.08(6)	0.361	3245.2±278.76(5)	0.104	4838.3±768.11(5)	0.233
**F1:♂:HFD:10**	**0**	1.2±0.45(8)	0.303	6563.7±892.98(8)	0.144	4629.6±776.32(8)	0.687	5155.3±486.5(8)	0.016
**F1:♂:HFD:10**	**0.5**	1±0.22(6)	0.184	5860.9±808.57(7)	0.221	4960.7±1118.85(7)	0.514	4706±590.63(7)	0.12
**F1:♂:HFD:10**	**5.0**	1.2±0.27(8)	0.944	7007.1±1395.99(8)	0.42	5024.2±986.12(8)	0.422	5588±530.09(8)	0.121
**F1:♂:HFD:24**	**0**	1.8±0.48(5)	0.286	7193±565.63(6)	0.148	2382.5±700.31(5)	0.909	4844±154.41(6)	0
**F1:♂:HFD:24**	**0.5**	1.7±0.23(5)	0.662	6508±261.47(6)	0.056	2259±255.45(6)	0.67	3778±291.71(6)	0
**F1:♂:HFD:24**	**5.0**	1.4±0.21(3)	0.128	6771.6±774.45(6)	0.223	2303.6±232.23(3)	0.821	5280.8±142.59(3)	0.016
**F1:♀:LFD:10**	**0**	1.1±0.19(6)	0.023	7346.6±735.33(6)	0.132	3660.9±380.21(6)	0.176	4767.5±687.4(6)	0.005
**F1:♀:LFD:10**	**0.5**	1±0.16(7)	0.48	6689.5±866.86(9)	0.127	3956.1±258.19(8)	0.145	4528.6±1281.89(8)	0.68
**F1:♀:LFD:10**	**5.0**	1.4±0.33(10)	0.063	6508.1±734.13(10)	0.051	4011.9±414.49(10)	0.074	6265.9±1033.23(10)	0.012
**F1:♀:LFD:24**	**0**	1.1±0.13(5)	0.282	6653.8±1524.36(5)	0.274	3300.1±143.68(4)	0.011	5365±388.71(5)	0
**F1:♀:LFD:24**	**0.5**	0.9±0.45(5)	0.219	5746.3±820.48(5)	0.174	4008.1±353.02(5)	0.007	3840±165.61(5)	0
**F1:♀:LFD:24**	**5.0**	1.1±0.13(7)	0.847	6658.7±431.05(6)	0.994	3415.9±379.93(7)	0.587	5242.6±707.7(7)	0.691
**F1:♀:HFD:10**	**0**	1.2±0.4(7)	0.09	7190±980.53(7)	0.751	3564.4±454.75(7)	0.243	5139.2±502.28(7)	0.255
**F1:♀:HFD:10**	**0.5**	1±0.27(9)	0.128	7315±1019.91(8)	0.976	4084.8±392.79(8)	0.099	5664.9±2289.64(9)	0.485
**F1:♀:HFD:10**	**5.0**	1.3±0.27(10)	0.64	9678.1±12111.31(10)	0.523	3896.4±758.14(10)	0.261	6352±830.63(10)	0.108
**F1:♀:HFD:24**	**0**	1.4±0.23(4)	0.118	6780.6±789.74(4)	0.428	3098.2±326.52(3)	0.749	4274.6±806.79(4)	0.005
**F1:♀:HFD:24**	**0.5**	1.2±0.21(5)	0.087	5993.2±1248.08(5)	0.309	3207.5±135.14(5)	0.519	4070.4±272.05(5)	0.664
**F1:♀:HFD:24**	**5.0**	1.2±0.09(5)	0.057	7054.2±1104.74(2)	0.777	3252±NA(1)	0.565	5774±845.6(5)	0.007
**Exposure**			0.002		NS		NS		<0.001
**Diet (Low fat vs. high fat)**			<0.001		NS		NS		NS
**Exposure: Diet**			0.009		NS		NS		NS

Copper ANOVA Summary				
	Pr(>F)			
	**Liver**	**Kidney**	**Heart**	**Blood**
**F0**				
Cadmium Concentration	0.189	0.734	0.323	0.355
Sex	0	0.008	0.826	0
Cadmium Concentration:Sex	0.03	0.792	0.642	0.151
Residuals	-	-	-	-
**F1**				
Cadmium Concentration	0.106	0	0.31	0.002
Sex	0.001	0.101	0.624	0.611
Diet	0.07	0.412	0.539	0
Exposure Time	0	0	0.919	0.008
Cadmium Concentration:Sex	0.072	0.036	0.993	0.076
Cadmium Concentration:Diet	0.936	0.121	0.857	0.177
Sex:Diet	0.953	0.183	0.223	0.009
Cadmium Concentration:Exposure Time	0.574	0.054	0.914	0.002
Sex:Exposure Time	0	0.686	0.127	0.024
Treat:Exposure Time	0	0.349	0.302	0
Cadmium Concentration:Sex:Diet	0.81	0.485	0.448	0.877
Cadmium Concentration:Sex:Exposure Time	0.799	0.825	0.834	0.522
Cadmium Concentration:Diet:Exposure Time	0.405	0.216	0.413	0.385
Sex:Diet:Exposure Time	0.019	0.244	0.871	0.179
Cadmium Concentration:Sex:Diet:Exposure Time	0.899	0.659	0.954	0.852
Residuals	-	-	-	-

Cobalt Concentration Summary									
		Blood		Heart		Liver		Kidney	
**Generation:Sex:diet:exposure time**	**Metal Concentration (ppm)**	**Concentration**	**p value**	**Concentration**	**p value**	**Concentration**	**p value**	**Concentration**	**p value**
**F1:♂:LFD:10**	**0**	0±0(7)	0.03	17.1±18.45(8)	0.37	18.1±3(8)	0.806	23.6±3.83(8)	0.003
**F1:♂:LFD:10**	**0.5**	0±0(8)	0.43	7.7±3.39(8)	0.682	17.4±3.9(8)	0.651	16±5.13(8)	0.001
**F1:♂:LFD:10**	**5.0**	0±0(7)	0.063	40.8±80.03(7)	0.324	18.5±3.14(7)	0.848	21.6±2.37(7)	0.339
**F1:♂:LFD:24**	**0**	0±0(5)	0.84	23.2±7.11(4)	0.419	18.4±5.15(4)	0.649	31.1±7.73(5)	0.036
**F1:♂:LFD:24**	**0.5**	0±0(4)	0.85	13.9±2.43(4)	0.566	17.9±1.86(4)	0.858	16.3±4.7(4)	0.012
**F1:♂:LFD:24**	**5.0**	0±0(5)	0.693	33.6±32.78(6)	0.491	16.3±2.5(5)	0.395	25.8±8.47(5)	0.277
**F1:♂:HFD:10**	**0**	0±0(8)	0.508	9.4±4.22(8)	0.816	13.1±3.54(8)	0.357	20.1±4.46(8)	0.086
**F1:♂:HFD:10**	**0.5**	0±0(6)	0.683	7.5±6.47(7)	0.529	14.6±4.43(7)	0.416	15.6±3.56(7)	0.029
**F1:♂:HFD:10**	**5.0**	0±0(8)	0.254	8.6±6.18(8)	0.779	11.9±2.44(8)	0.514	17.7±2.89(8)	0.204
**F1:♂:HFD:24**	**0**	0±0(5)	0.795	19.3±3.81(6)	0.001	10.5±5.2(5)	0.954	21.5±2.02(6)	0.165
**F1:♂:HFD:24**	**0.5**	0±0(5)	0.751	10.6±1.17(6)	0.002	9.8±2.51(6)	0.766	16.4±6.19(6)	0.08
**F1:♂:HFD:24**	**5.0**	0±0(3)	0.51	20.5±5.86(6)	0.631	10.1±1.22(3)	0.878	21.1±4.37(3)	0.925
**F1:♀:LFD:10**	**0**	0±0(6)	0.693	15.5±6.82(6)	0.138	20.1±7.01(6)	0.713	30.5±9.59(6)	0.079
**F1:♀:LFD:10**	**0.5**	0±0(7)	0.943	12.8±6.15(9)	0.376	19.9±6.23(8)	0.926	24.9±10.96(8)	0.372
**F1:♀:LFD:10**	**5.0**	0±0(10)	0.517	9.5±4.63(10)	0.054	18.1±4.07(10)	0.481	37.7±12.62(10)	0.231
**F1:♀:LFD:24**	**0**	0±0(5)	0.059	28±8.11(5)	0.001	22.5±4.03(4)	0.149	53.4±22.95(5)	0.548
**F1:♀:LFD:24**	**0.5**	0±0(5)	0.02	10.4±2.45(5)	0	23.4±3.06(5)	0.739	38.5±17.41(5)	0.287
**F1:♀:LFD:24**	**5.0**	0±0(7)	0.134	23±4.45(6)	0.159	19±4.06(7)	0.167	47.8±22.32(7)	0.661
**F1:♀:HFD:10**	**0**	0±0(7)	0.152	11.5±8.06(7)	0.693	13.3±4.83(7)	0.616	22.8±5.94(7)	0.781
**F1:♀:HFD:10**	**0.5**	0±0(9)	0.695	8±7.22(8)	0.412	17.1±5.77(8)	0.33	23.8±20.03(9)	0.88
**F1:♀:HFD:10**	**5.0**	0±0(10)	0.176	10.4±8.88(10)	0.775	15.2±9.62(10)	0.597	27±6.89(10)	0.52
**F1:♀:HFD:24**	**0**	0±0(4)	0.048	19.6±5.76(4)	0.185	20.6±5.76(3)	0.784	30.5±5(4)	0.202
**F1:♀:HFD:24**	**0.5**	0±0(5)	0.043	13.7±4.24(5)	0.145	20.9±1.47(5)	0.935	30.5±4.37(5)	1
**F1:♀:HFD:24**	**5.0**	0±0(5)	0.022	21.8±8.41(2)	0.668	23.4±NA(1)	0.52	41.8±16.3(5)	0.137
**Exposure**			0.008		NS		NS		0.001
**Diet (Low fat vs. high fat)**			NS		NS		<0.001		<0.001
**Exposure: Diet**			NS		NS		NS		NS

Cobalt ANOVA Summary				
	Pr(>F)			
	**Liver**	**Kidney**	**Heart**	**Blood**
**F0**				
Cadmium Concentration	0.818	0.256	0.195	0.441
Sex	0.376	0.492	0.003	0.288
Cadmium Concentration:Sex	0.779	0.408	0.424	0.748
Residuals	-	-	-	-
**F1**				
Cadmium Concentration	0.414	0.001	0.052	0.008
Sex	0	0	0.338	0.26
Diet	0	0	0.034	0.885
Exposure Time	0.464	0	0.035	0.027
Cadmium Concentration:Sex	0.43	0.35	0.165	0.42
Cadmium Concentration:Diet	0.548	0.223	0.39	0.966
Sex:Diet	0.136	0.116	0.214	0.761
Cadmium Concentration:Exposure Time	0.816	0.615	0.729	0.029
Sex:Exposure Time	0.002	0.008	0.742	0.903
Treat:Exposure Time	0.973	0.318	0.681	0.785
Cadmium Concentration:Sex:Diet	0.584	0.872	0.198	0.291
Cadmium Concentration:Sex:Exposure Time	0.899	0.952	0.684	0.131
Cadmium Concentration:Diet:Exposure Time	0.467	0.357	0.807	0.575
Sex:Diet:Exposure Time	0.063	0.611	0.681	0.83
Cadmium Concentration:Sex:Diet:Exposure Time	0.964	0.669	0.607	0.878
Residuals	-	-	-	-
